# High throughput profiling of the cotton bollworm *Helicoverpa armigera* immunotranscriptome during the fungal and bacterial infections

**DOI:** 10.1186/s12864-015-1509-1

**Published:** 2015-04-18

**Authors:** Guang-Hua Xiong, Long-Sheng Xing, Zhe Lin, Tusar T Saha, Chengshu Wang, Haobo Jiang, Zhen Zou

**Affiliations:** State Key Laboratory of Integrated Management of Pest Insects and Rodents, Institute of Zoology, Chinese Academy of Sciences, Beijing, 100101 China; Department of Entomology, University of California Riverside, Riverside, CA 92521 USA; Key Laboratory of Insect Developmental and Evolutionary Biology, Institute of Plant Physiology and Ecology, Shanghai Institutes for Biological Sciences, Chinese Academy of Sciences, Shanghai, 200032 China; University of Chinese Academy of Sciences, Beijing, 100049 China; Department of Entomology and Plant Pathology, Oklahoma State University, 127 NRC, Stillwater, OK 74078 USA

**Keywords:** *Helicoverpa armigera*, Fungal infection, Bacterial challenge, Innate immunity, RNA-seq, Fat body, Hemocytes

## Abstract

**Background:**

Innate immunity is essential in defending against invading pathogens in invertebrates. The cotton bollworm, *Helicoverpa armigera* (Hübner) is one of the most destructive lepidopteran pests, which causes enormous economic losses in agricultural production worldwide. The components of the immune system are largely unknown in this insect. The application of entomopathogens is considered as an alternative to the chemical insecticides for its control. However, few studies have focused on the molecular mechanisms of host-pathogen interactions between pest insects and their pathogens. Here, we investigated the immunotranscriptome of *H. armigera* larvae and examined gene expression changes after pathogen infections. This study provided insights into the potential immunity-related genes and pathways in *H. armigera* larvae.

**Results:**

Here, we adopted a high throughput RNA-seq approach to determine the immunotranscriptome of *H. armigera* larvae injected with buffer, fungal pathogen *Beauveria bassiana*, or Gram-negative bacterium *Enterobacter cloacae*. Based on sequence similarity to those homologs known to participate in immune responses in other insects, we identified immunity-related genes encoding pattern recognition receptors, signal modulators, immune effectors, and nearly all members of the Toll, IMD and JAK/STAT pathways. The RNA-seq data indicated that some immunity-related genes were activated in fungus- and bacterium-challenged fat body while others were suppressed in *B. bassiana* challenged hemocytes, including the putative IMD and JAK-STAT pathway members. Bacterial infection elevated the expression of recognition and modulator genes in the fat body and signal pathway genes in hemocytes. Although fat body and hemocytes both are important organs involved in the immune response, our transcriptome analysis revealed that more immunity-related genes were induced in the fat body than that hemocytes. Furthermore, quantitative real-time PCR analysis confirmed that, consistent with the RNA-seq data, the transcript abundances of putative PGRP-SA1, Serpin1, Toll-14, and Spz2 genes were elevated in fat body upon *B. bassiana* infection, while the mRNA levels of defensin, moricin1, and gloverin1 were up-regulated in hemocytes.

**Conclusions:**

In this study, a global survey of the host defense against fungal and bacterial infection was performed on the non-model lepidopteran pest species. The comprehensive sequence resource and expression profiles of the immunity-related genes in *H. armigera* are acquired. This study provided valuable information for future functional investigations as well as development of specific and effective agents to control this pest.

**Electronic supplementary material:**

The online version of this article (doi:10.1186/s12864-015-1509-1) contains supplementary material, which is available to authorized users.

## Background

The cotton bollworm *Helicoverpa armigera*, is a destructive and highly polyphagous insect pest in Asia, Europe, Africa, and Australia. It causes serious damage to cotton, sorghum, corn and several other crops. Further aggravating the situation, this pest is spreading geographically by invading newer territory. Recently, their presence outside the Asian continent has been confirmed with reports of establishment of this species in Brazil [1]. Primary methods of controlling this devastating agricultural pest include traditional spraying of pesticides and cultivation of transgenic pest resistant crop varieties developed in the recent past. However, apart from causing environmental pollution these methods also lead to resistance development in pests, thus necessitating immediate development of novel biological control methods. Entomopathogenic fungi like *Beauveria bassiana* has been widely considered as a potent eco-friendly bio-control agent [2]. The potential of *B. bassiana* as an effective suppressant of *H. armigera* and other lepidopteran pests is under consideration [3]. Before *B. bassiana* kills the pest, it needs to invade and overcome the host immune responses that have not been well understood in *H. armigera*.

The invasion of pathogens and parasites into an insect is defended by innate immune system, a strong universal defense mechanism shared by both vertebrates and invertebrates [4]. This system consists of initial physical barriers to pathogen penetration followed by humoral and cellular response against subsequent microbial infection. While the cellular response involves phagocytosis, encapsulation, and nodule formation by the circulating hemocytes, the humoral response refers to the process of melanization and the production of immune effector molecules, which are mainly produced in the fat body. Anti-bacterial immunity depends on two principal signaling pathways, Toll and IMD, which are conserved across various insect species indicating their central importance throughout arthropod evolution [5,6]. These pathways are activated by the binding of pattern recognition receptors (PRRs) such as peptidoglycan recognition protein (PGRP), β-1,3-glucan recognition protein (βGRP), C-type lectin (CTL) to pathogen associated molecular pattern (PAMP) on the surface of invading microorganisms [4]. Specifically, Gram-negative bacteria trigger the IMD pathway leading to an acute response, while Gram-positive bacteria and fungi activate the Toll pathway eliciting a more sustained and complex reaction [7]. Both of these signaling pathways result in the induction of various NF-кB transcription factors, which then drive a comprehensive synthesis of immune effectors including antimicrobial peptides (AMPs) [7]. Like immune signaling pathways, melanization which results from the activation of prophenoloxidase (PPO), is also considered as an universal immune response against bacteria in insects [8]. It involves melanin synthesis, sequestering and killing the invading microorganisms or parasites [8].

After invading the host, *B. bassiana* triggers the cellular and humoral immune mechanism of the host resulting in melanized nodules and activation of Toll pathway [9,10]. The Toll signaling pathway is mediated by an extracellular protease cascade composed of a series of clip-domain serine protease (cSP), which helps to amplify the initial recognition signal [7]. Several immune molecules like GNBP3, Persephone, and Drosomycin are involved in the fungus activated Toll pathway [9]. Besides the Toll pathway, melanization is also implicated to play a role in antifungal immunity. *B. bassiana* infection of mosquito *Aedes aegypti* enhances the cleavage of hemolymph PPO and its conversion to phenoloxidase (PO), the rate limiting enzyme in melanogenesis [11]. Similarly, induced melanization in another mosquito *Anopheles gambiae* retarded the growth and dissemination of *B. bassiana* [10]. However, the thorough understanding of interaction between melanization and fungal challenge is largely unclear.

*B. bassiana* parasitizes a wide range of hosts including *H. armigera* and other lepidopteran pests. Most studies on insect immunity were performed in model organisms, such as *Drosophila melanogaster* [3], *Manduca sexta* [8], and *Tenebrio molitor* [12]. Systematic analysis of immunity-related genes has been conducted in several other insects including *A. gambiae*, *A. aegypti*, *Bombyx mori*, *Apis mellifera* and *Tribolium castaneum*, whose genome sequences are available. It is only recently that *H. armigera* immunity started to attract some researchers’ attentions [13]. Fourteen genes involved in the *H. armigera* interaction with *Bacillus thuringiensis* and *Autographa californica* multiple nucleocapsid nucleopolyhedrovirus were obtained [14]. The expression and characterization of CTLs and PGRPs in response to pathogen were also reported [15-17]. However, *H. armigera* genome is still unavailable and its immune response to *B. bassiana* remains largely unexplored, restricting its further development and adoption as a biological agent in the control of lepidopteran pests.

More interestingly, systematic survey on *H. armigera* is of great importance for understanding the origin and evolution of immune systems, which is a challenging task since genetic backgrounds of non-model insects are unclear, let alone expression profiling. In this work, RNA-seq was employed to perform immunotranscriptome analysis of *H. armigera* larval hemocytes and fat body in response to the challenges of entomopathogenic fungi *B. bassiana* and Gram-negative bacteria *Enterobactor cloacae*. In an attempt to gain insights into the *H. armigera* immune response against pathogens, we characterized 233 such genes and divided them into pattern recognition, signal transduction, execution, and cellular responses. Hierarchical clustering analyses of differentially expressed gene (DEG) cohorts indicated that the fungi and bacteria elicited distinct expression profiles of genes in hemocytes and fat body. These results provide an overview of the tissue-specific expression profiles in response to infections and a platform for further exploring the molecular basis of host antimicrobial response.

## Results and discussion

### Immunization, sequencing assembly, and gene identification

**Figure 1:**
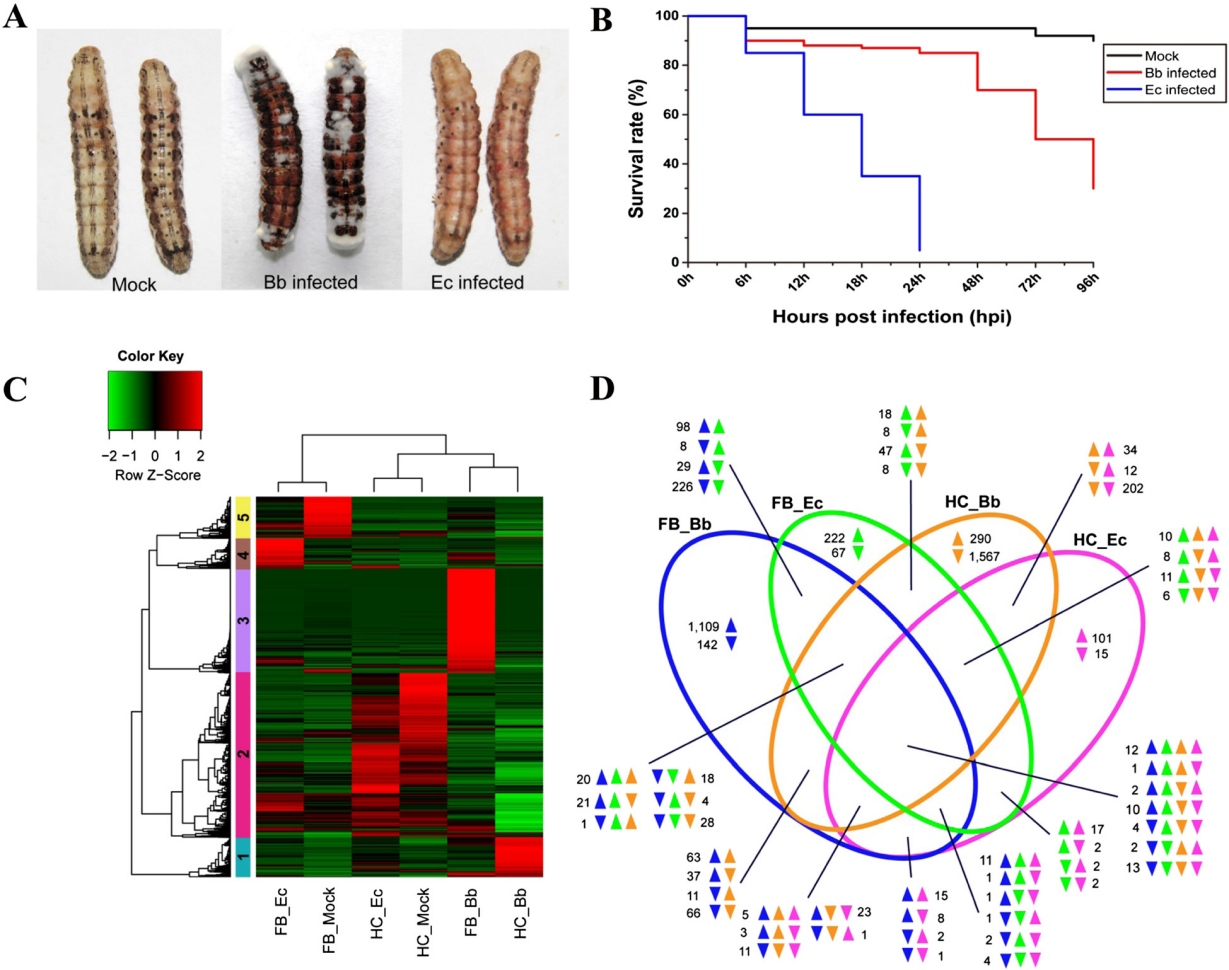
Distinct pathogenicity and transcriptomic change elicited by *B. bassiana* and *E. cloacae*. **(A)** Different phenotypes of the cotton bollworm larvae infected with *B. bassiana* (Bb) and *E. cloacae* (Ec). The *B. bassiana*-infected larvae were covered with white hyphae at 72 h post infection and then became rigid before death. However, *E. cloacae*-infected larvae turned light red at 12 h post infection and became gradually liquefied before death. **(B)** Survival curves of *H. armigera* larvae infected by Bb (red line) and Ec (blue line) in comparison with the control group (black line). Three repeats, p < 0.01. **(C)** Hierarchical clustering analysis of DETs in fat bodies and hemocytes of *H. armigera* larvae infected by Bb 48 h post infection and Ec 6 h post infection, respectively. The transcript levels significantly (p < 0.001) increased (>2 fold) and decreased (<0.5 fold) under at least two experimental conditions. The heatmap is divided into five discrete clusters and color coded on the left. Cluster 1 (cyan, induced) and Cluster 2 (magenta, suppressed) in hemocytes from Bb infected larvae; cluster 3 (purple) and 4 (brown) induced in fat body of Bb and Ec infected larvae, respectively; cluster 5 (yellow) genes suppressed in fat body of Bb and Ec infected larvae. FB_Mock, control fat body; FB_Bb, fungus-induced fat body; FB_Ec, bacterium-induced fat body; HC_Mock, control hemocytes; HC_Bb, fungus-induced hemocytes; HC_Ec, bacterium-induced hemocytes. **(D)** A Venn diagram showing the shared and unique DETs in fat body and hemocytes, induced by the fungal and bacterial infection. The overlapping regions represent genes that are concomitantly regulated in two, three or four samples. The directions of transcript level changes are indicated by upward- and downward-pointing arrows. Blue, green, brown and purple represent Bb-fat body, Ec-fat body, Bb-hemocytes, and Ec-hemocytes, respectively.

Most immunity studies on lepidopteran insects use dead microbes, aiming at deciphering biochemical mechanisms. In contrast, dipteran insects such as *Drosophila* and mosquitoes are often injected with live pathogens, which eventually kill the hosts. In order to decipher host-pathogen interactions and develop better pest control methods, we performed septic injury on day 2 fifth instar larvae using two entomopathogens, *B. bassiana*, and *E. cloacae*, which elicit an inefficient immune response compared with the challenge by dead pathogens. The immunity-related genes based on their expression profiles in fat body or hemocytes were obtained through comparison of the immune challenged transcriptomes. These two entomopathogenic agents had distinct morphological characteristics on plates and under light microscope (in Additional file 1: Figure S1). *E. cloacae* injected larvae turned light red in color at 12 h after infection and were almost completely liquefied before death by 24 h (Figure 1A), as compared with the light brown color in larvae that were injected with the phosphate buffered saline (PBS). Fungal infection on the other hand was slower, with almost 85% survival rate at 24 h post injection as compared to the 15% of that of bacteria (Figure 1B). *B. bassiana* injected larvae turned dark brown and were finally covered with white conidia and hyphae at 72 h post infection, leading to the death of the insect. These results suggested a distinct pathogenicity of *B. bassiana* and *E. cloacae* toward the cotton bollworm.

In *D. melanogaster* and mosquitoes, immunity-related genes regulated by IMD pathway showed an acute expression pattern involved in the anti-bacterial responses, whereas Toll pathway displayed sustained activation of immunity-related genes against fungal infection [5,18]. To gain detailed information about the *H. armigera* transcriptome, the hemocytes and fat body samples were collected from *E. cloacae* infected larvae at 6 h and *B. bassiana* infected larvae at 48 h, respectively. Six cDNA libraries were constructed from the RNA samples extracted from control fat body (FB_Mock), fungus-induced fat body (FB_Bb), bacterium-induced fat body (FB_Ec), control hemocytes (HC_Mock), fungus-induced hemocytes (HC_Bb), and bacterium-induced hemocytes (HC_Ec) (in Additional file 1: Figure S2). These libraries were sequenced on an Illumina Hiseq 2000 system. After removal of adaptor sequences and low-quality reads (Q < 20), these libraries yielded 72.6, 79.2, 65.6, 80.1, 46.7, and 80.4 million high-quality reads with 7.54, 7.70, 5.90, 7.53, 8.25, and 7.45 Gb data, respectively (in Additional file 2: Table S1). All the reads were *de novo* assembled into 57,588, 61,452, 55,394, 60,873, 42,073, and 58,262 contigs with average lengths of 1,102, 1,139, 1,034, 1,128, 995, and 1,188 nt, respectively [19,20]. Pooled clean reads from the six libraries and three others (*E. cloacae*, *B. bassiana*, and PBS injected 2nd instar larvae), totally yielded 150,606 contigs (average size: 1,124 nt) (in Additional file 2: Table S1).

To predict functions, all the obtained unigenes were annotated according to BLASTX searches against the non-redundant sequence database resulting 60,458 (43.2%) of them displaying homology to the known proteins (E-value < 1e-5) (in Additional file 1: Figure S3) [21]. For species distribution, nearly 17,000 (11.2%) annotated unigenes were homologous to *B. mori*, followed by *Danaus plexippus* (6.6%). Only fewer transcripts were matched to the ones from *H. armigera* (0.6%), probably due to the unavailability of its genome sequences (in Additional file 1: Figure S3). Additionally, 713 transcripts with high similarity to non-coding RNAs by homology search were obtained (in Additional file 3: Table S2) [22]. After removing redundant sequences and those shorter than 150 nt, we retained the unigenes with significant BLAST hits and generated a non-redundant dataset containing 37,694 sequences (average length: 1,711 nt) (in Additional file 2: Table S1). Length distribution of the *H. armigera* transcripts indicates that this final dataset has a high proportion of long transcripts (>2000 nt) and increases the chances of full length gene predictions. Based on the unigene information (*e.g*. gene ID, length, putative function) (in Additional file 4: Table S3), the immunity-related genes were identified and filtered out for further analysis. The detailed workflow from immune challenge to immunity-related gene identification is provided in a flowchart (in Additional file 1: Figure S2).

### Identification and functional classification of differentially expressed genes in response to *B. bassiana* and *E. cloacae* infections

To gain insights into the tissue-specific transcriptional changes in *H. armigera* larvae infected by *B. bassiana* and *E. cloacae*, we performed pairwise comparisons between libraries to identify the differentially expressed transcripts (DETs) (in Additional file 1: Figure S4A) [23]. Relative to the control, transcripts with greater than 2-fold change and p value less than 0.001 were considered as differentially expressed. The complete list of DETs with FPKM (fragments per kilobase of transcript per million) values (in Additional file 5: Table S4) revealed that more unigenes exhibited remarkable changes in mRNA levels in fat body (530 up- and 415 down-regulated) than in hemocytes (235 up- and 320 down-regulated) 6 h after *E. cloacae* injection (in Additional file 1: Figure S4B) indicating a tissue-specific affect. More pronounced changes occurred to the larvae 48 h after *B. bassiana* injection. Compared to the control group, there were 2,014 (1,469 up- and 545 down-regulated) transcripts that were significantly changed in the fat body elicited by *B. bassiana*, while more transcripts (496 up- and 2081 down-regulated) suppressed in hemocytes were observed.

In order to compare gene expression levels in hemocytes and fat body in response to the pathogen invasion, we performed hierarchical clustering of DETs and identified five discrete clusters showing expression trends relevant to the infection. The 487 and 2,015 genes in clusters 1 and 2 had the highest and the lowest RNA levels in hemocytes from *B. bassiana* infected larvae, respectively, among all the experimental conditions (Figure 1C and in Additional file 5: Table S4). Clusters 3 (1,280 genes) and 4 (370 genes) showed the most significant mRNA level increase in fat body after the fungal and bacterial infection, respectively. Furthermore, cluster 5 represented gene cohorts significantly suppressed in hemocytes and fat body after the infections. Consistent with the information revealed by hierarchical clustering, a Venn diagram analysis indicated that 1,251 transcripts were exclusively regulated in the fungus challenged fat body (1,109 up- and 142 down-regulated), while 1,857 other transcripts were specifically regulated in the fungus challenged hemocytes (290 up- and 1,567 down-regulated) (Figure 1D). In comparison, fewer gene transcripts (222 + 67 + 101 + 15) were exclusively regulated in the two tissues after the bacterial infection. Considering that the gene repertoire of fat body had small overlap with that of hemocytes (Figure 1B), we speculate that tissue type instead of the pathogen is more important in regulating the gene transcription under the same experimental condition. Besides, the data showed consistent up-regulation of 12 genes in both fat body and hemocytes after the fungal and bacterial infection and most of them were immunity-related.

**Figure 2:**
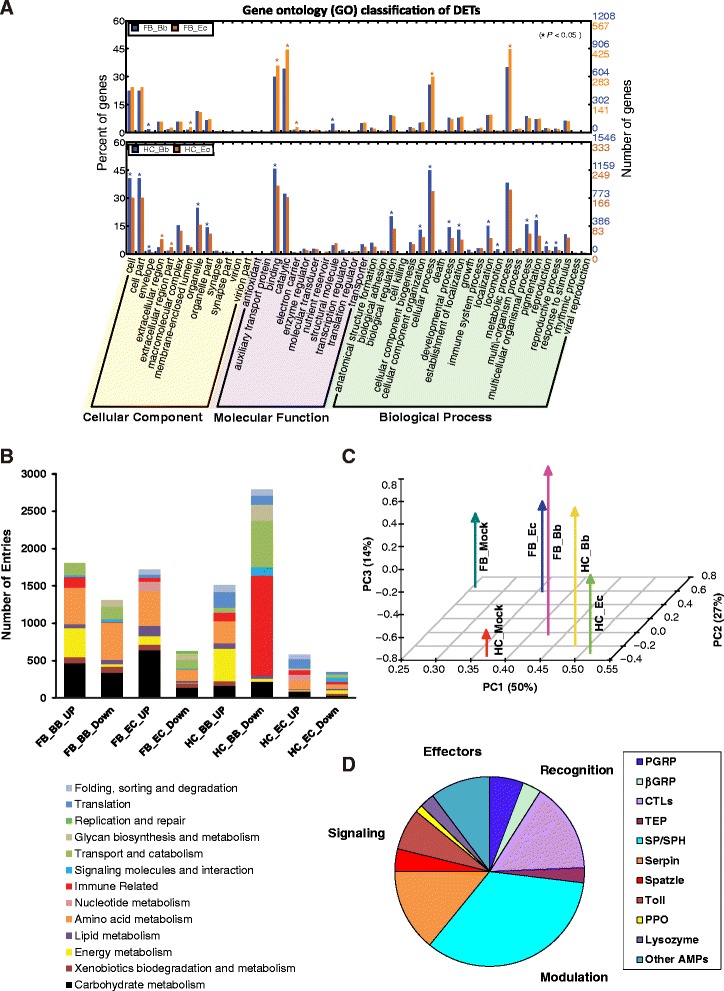
Comparative transcriptome analysis and identification of immunity-related genes in *H. armigera*. **(A)** Gene ontology (GO) annotation of DETs in the *H. armigera* transcriptome. Enriched GO analysis (* for p < 0.05) of DETs between *B. bassiana* (blue) and *E. cloacae* (brown) infections are performed by pairwise comparison with the corresponding control group of fat body (upper panel) or hemocytes (lower panel). Level 2 GO assignments are made in terms of cellular components, molecular functions, and biological processes. The number of gene transcripts assigned to each GO term is shown on the right y-axis, and its percentage of the total number of transcripts is on the left y-axis. **(B)** Distribution of KEGG functional groups within up- and down-regulated gene cohorts in fat body and hemocytes from the Bb and Ec-infected larvae. The bar chart corresponds to the matched entries of DETs in their own functional category. **(C)** Principal component analysis (PCA) analysis of global gene expression in hemocytes and fat body in response to the infections. Six samples were analyzed, including Bb-affected fat body (FB_Bb) and hemocytes (HC_Bb), Ec-affected fat body (FB_Ec) and hemocytes (HC_Ec), control fat body (FB_Mock) and hemocytes (HC_Mock). **(D)** Distribution of *H. armigera* immunity-related transcripts in categories of recognition, signaling, regulation, and effectors.

To analyze the potential functions of all identified DETs and the corresponding pathways involved, the Gene Ontology (GO) enrichment and KEGG analysis were further performed for DETs [24,25]. Functional classification of all unigenes was determined by GO assignment. In the gene repertoire of fat body, more DETs from the *E. cloacae* elicited transcriptome were assigned binding and catalytic activities in the category of molecular function and cellular and metabolic processes in biological process (Figure 2A). In contrast, more DETs from *B. bassiana* elicited hemocyte transcriptome were enriched in the following GO terms: cell, cell part, organelle, and organelle part in cellular component, binding in molecular function, and biological regulation and cellular process in biological process. We also used KEGG classification to analyze putative functions of the *B. bassiana* and *E. cloacae* induced DETs, which were categorized into thirteen functional groups based on the biological processes and molecular functions [26]. There was a remarkable tissue related disparity among the major functional gene groups between the fungi and bacteria induced transcriptomes (Figure 2B). Except in *E. cloacae* elicited hemocytes, a large proportion of up-regulated gene repertoire encoded enzymes involved in carbohydrate, energy, transport and catabolism, and immunity-related processes. Other notable functional gene groups were xenobiotics, glycan biosynthesis and metabolism, nucleotide metabolism, folding, sorting and degradation. In particular, such gene transcripts formed the largest group that were down-regulated in hemocytes after *B. bassiana* infection, followed by genes involved in replication and repair. We performed the principal component analysis (PCA) to compare changes in the transcriptomes and found clear variation between fat body and hemocyte samples (Figure 2C). Another variation exists between pathogen treated and control groups, whereas Ec or Bb treated samples are mostly similar. It appears that the difference between the tissues was much more significant than that between different pathogens.

Taken together, our results clearly indicated that global gene expression profiles varied among different tissues and treatments (Figures 1 and 2). While tissue is the major determinant, other factors also have minor but significant impacts on the transcriptome. The distinct GO and KEGG distribution of up- and down-regulated gene cohorts showed similar trends. Detailed expression profiling of the immunity-related genes was still required for further investigation of tissue-specific immune responses against *B. bassiana* and *E. cloacae* infection.

For in-depth analysis of the interactions between *H. armigera* and pathogens, we annotated the insect defense genes and examined their phylogenetic relationships. By sequencing the tissues that were enriched in the immune-related transcripts at sufficient depth, followed by the BLAST search [27], we were able to detect 233 immunity-related genes (Figure 2D, in Additional file 6: Table S5). This number was significantly higher when compared with other insects from holometabolous orders, such as *A. mellifera*, but similar to that in *B. mori*. This result indicated that *H. armigera*, well adapted to various plant hosts and environment, has a sizable repertoire of genes related to the cellular and humoral immune responses against the wounding and infection.

### Comparative analysis of genes for immune signal recognition

When pathogens infect insects, multiple PRRs may recognize conserved determinants (*e.g*. lipopolysaccharide, peptidoglycan, lipoteichoic acid, β-1,3-glucan) on the pathogen surface to trigger host defense reactions [28]. While some PRRs are constitutively expressed as surveillance molecules at certain levels in hemolymph, others are synthesized in response to the entry of microorganisms. Upon binding to their target molecules, PRRs undergo a conformational change required for recruiting plasma factors and hemocytes that eliminate the invading pathogens [8], finally leading to PPO activation and synthesis of immune effectors (*e.g*. AMPs) via immune signaling pathways [8]. The *H. armigera* immunotranscriptome has at least 41 PRR transcripts, including 9 PGRPs, 5 βGRPs, 24 CTLs, and 3 galectins (in Additional file 6: Table S5, and in Additional file 7: Table S6).

**Figure 3:**
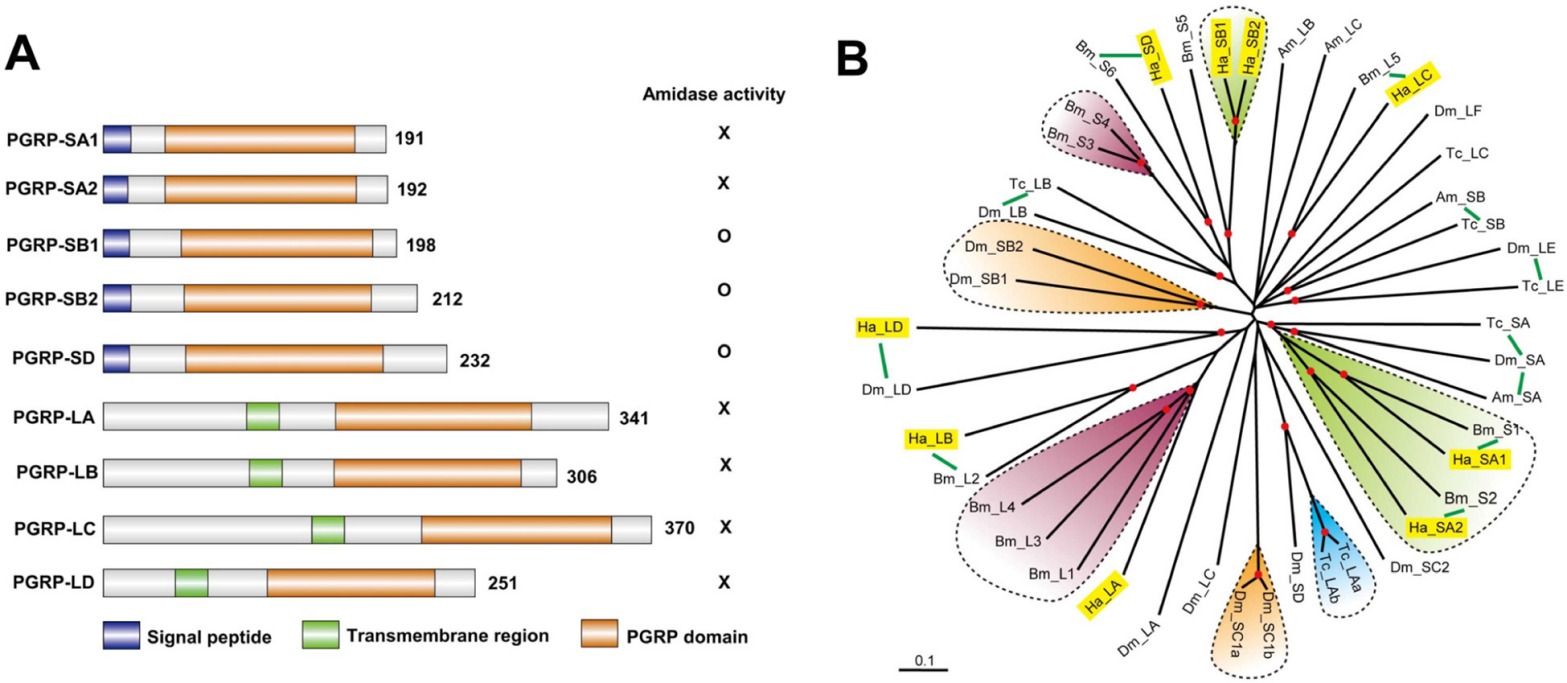
The PG recognition proteins of *H. armigera*. **(A)** Schematic representations of the *H. armigera* PGRP domain structures. Lengths of the amino acid sequences are indicated. **(B)** Phylogenetic analysis of PGRPs. The amino acid sequences of 9 *H. armigera* (Ha), 7 *T. castaneum* (Tc), 13 *D. melanogastor* (Dm), 11 *B. mori* (Bm), and 4 *A. mellifera* (Am) PGRPs are compared. Scale bar, 0.1 substitutions per site. The tree shows that HaPGRP and BmPGRPs form good orthologous groups, except for HaPGRP-LD. Red dots at nodes indicate bootstrap values greater than 800 from 1,000 trials. The putative 1:1 or 1:1:1 orthologs were connected by green lines. HaPGRP-LA, −LB, −LC, and -LD contain a transmembrane domain, while HaPGRP-SA1, −SA2, −SB1, −SB2, and -SD have a signal peptide. HaPGRP-SB1,-SB2, and -SD contain the key residues of the amidase activity that hydrolyzes PGs.

PGRPs play a pivotal role in activating insect innate immunity by binding to Lys- and diaminopimelate-type peptidoglycans in bacterial cell walls [7]. The members of this protein family are characterized by the presence of a conserved C-terminal PGRP domain (approximately 165 aa), which is homologous to bacteriophage and bacterial type 2 amidases [29]. PGRPs can be further sub-grouped into short (S) and long (L) forms based on their length and the presence of transmembrane domain. There are 13, 7, 12, and 3 PGRPs in the genomes of *D. melanogaster*, *A. gambiae*, *B mori* and *A. mellifera*, respectively. We have identified nine putative PGRP transcripts in *H. armigera* transcriptome and have named them as *HaPGRP-SA1*, −*SA2*, −*SB1*, −*SB2*, −*SD*, −*LA*, −*LB*, −*LC*, −*LD*, in accordance with PGRP nomenclature from other insects (Figure 3, in Additional file 6: Table S5) [17]. The mRNA abundances of *H. armigera* PGRP-SA1, −SB1, and -SD increased in response to the bacterial infection, and their roles in bacteria agglutination and growth inhibition have been characterized as well [30]. Multiple sequence alignment suggested that *PGRP-SB1*, −*SB2*, −*SD* contains all the five active site residues (H, Y, H, T, C) essential for amidase activity, indicating that they can not only bind but also degrade peptidoglycan (in Additional file 1: Figure S5). Based on their homology to BmPGRP-S1 and S5, a putative role of HaPGRP-SA1, −SB1 and SB2 in regulation of PPO activation can be predicted [31,32]. All the S form HaPGRPs have an N-terminal signal peptide and are expected to be secreted outside the cell (Figure 3). On the other hand, HaPGRP-LA, −LB, −LC, −LD contains a transmembrane region, potentially serving as peptidoglycan receptors triggering immune signaling pathways. There existed no *PGRP-LE* in *H. armigera* with similarity to that of *A. gambiae*, *B. mori*, and *A. mellifera*, while *PGRP-LD* has not been identified in *B. mori* (Figure 3B).

**Figure 4:**
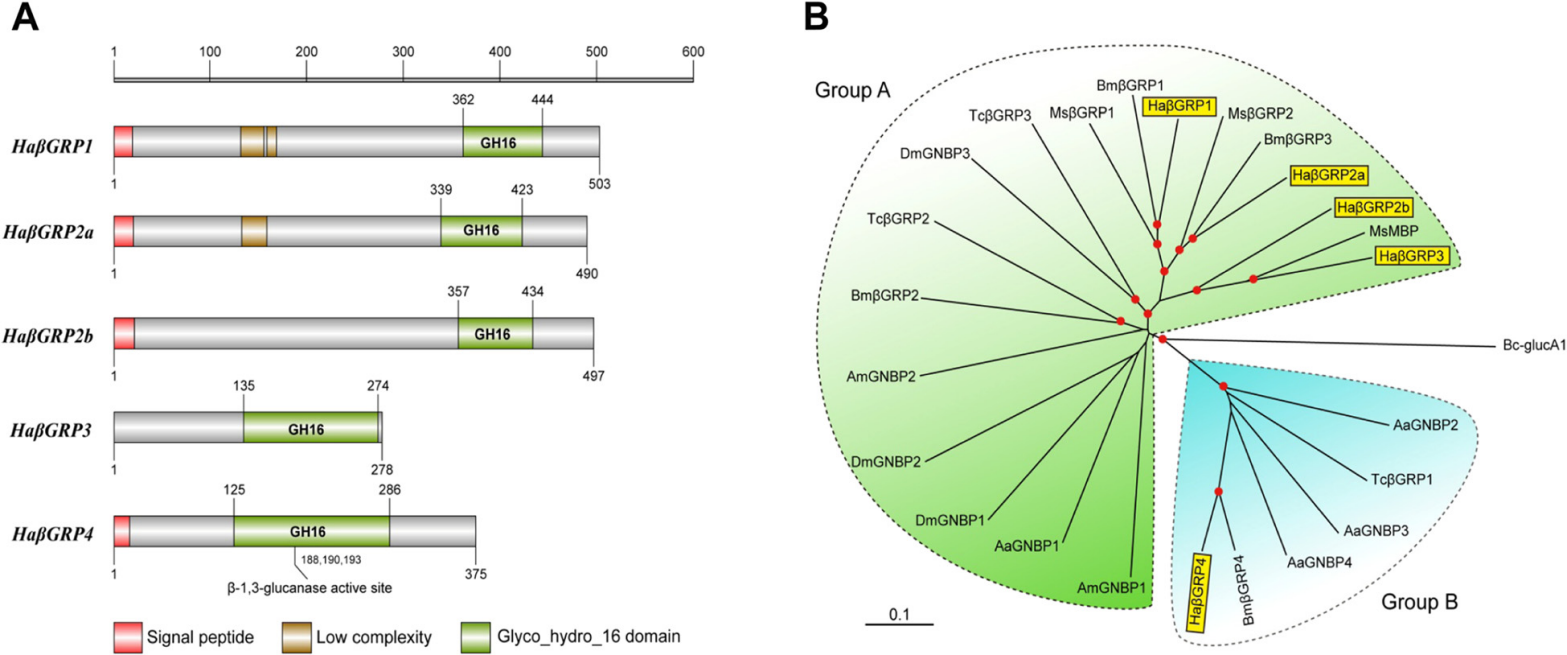
The β-1,3-glucanase related proteins (βGRPs) of *H. armigera*. **(A)** Schematic representations of the domain structure of *H. armigera* βGRPs. Lengths of the amino acid sequences are indicated. **(B)** Phylogenetic relationships of the βGRPs. Sequences of the βGRP family members from 5 *H. armigera* (Ha), 3 *T. castaneum* (Tc), 3 *D. melanogaster* (Dm), 4 *B. mori* (Bm), 2 *A. mellifera* (Am) PGRPs, 4 *A. aegypti* (Aa), and 2 *M. sexta* (Ms) are aligned to build the tree. Scale bar, 0.1 substitutions per site. *Bacillus circulans* (Bc) β-1,3-glucanase is included as an out-group.

βGRPs belong to another family of PRRs, which have a β-1, 3-glucanase-like domain close to the carboxyl terminus [28]. They could bind to β-1, 3-glucan (a fungal cell wall component) but lack the hydrolase activity, due to amino acid substitutions in the catalytic center. Other members of the family are referred to as GNBPs (Gram-negative bacteria-binding proteins). It has been reported that the mRNA level of *B. mori* GNBP was significantly elevated in response to bacterial infection [33]. In our transcriptome, five βGRP transcripts are identified and designated as *H. armigera* βGRP1 through 4, all of which may be secreted into hemolymph except βGRP3 (Figure 4, in Additional file 6: Table S5). The phylogenetic analysis of insect βGRP gene family revealed segregation into two major evolutionary groups – one retaining the key residues for glucanase activity (Group B) and the other lacking such residues (Group A) [34]. *H. armigera* and *B. mori* βGRP1, 2a, and 4 share a 1:1 orthology, while *H. armigera* βGRP-3 is orthologous to *M. sexta* MBP [34]. βGRP1-3 belong to a lepidopteran specific clade probably arising as a result of gene duplication (Figure 4). Based on the studies in *M. sexta* and orthologous relationships, we suggest that *H. armigera* βGRP1 and 2a bind to β-1,3-glucan to trigger a serine protease cascade for PPO activation. The close relationships among *H. armigera* βGRP1, 2a, 2b, 3 and *D. melanogaster* GNBP3 also indicate that the two β-1,3-glucanase-related proteins could be involved in antifungal immunity of *H. armigera*.

**Figure 5:**
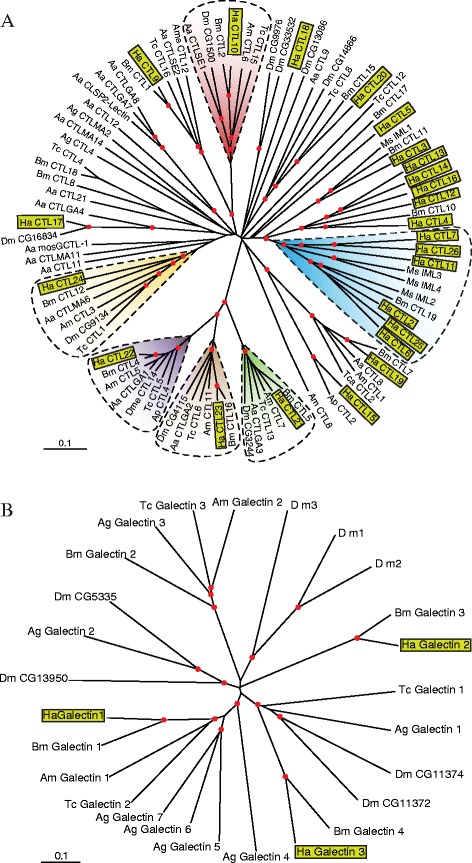
The C-type lectins (CTLs) and galectins of *H. armigera*. **(A)** Phylogenetic analysis of CTLs. The amino acid sequences of 24 *H. armigera* (Ha), 10 *T. castaneum* (Tc), 10 *D. melanogaster* (Dm), 14 *B. mori* (Bm), 4 *M. sexta* (Ms), 18 *A. aegypti* (Aa), 2 *A. gambiae* (Ag), 2 *Acyrthosiphon pisum* (Ap) and 8 *A. mellifera* (Am) CTLs are examined. Scale bar, 0.1 substitutions per site. **(B)** Phylogenetic analysis of galectins. The amino acid sequences of 3 Ha, 3 Tc, 7 Dm, 4 Bm, 7 Ag and 2 Am galectins are compared. Scale bar, 0.1 substitutions per site.

C-type lectins (CTLs) are a sub-group of carbohydrate binding lectin proteins that require the presence of calcium for its association with various types of sugar moieties like mannose and galactose. The protein family shows a considerable amount of diversity in terms of structure and function and can occur in membrane bound or soluble forms. Several insect CTLs have been implicated to play a role in host defense functioning as PRRs and eliciting multiple immune responses including PPO activation, hemocyte-mediated nodule formation, encapsulation and opsonization finally resulting in microbial clearance [35,36]. Previous studies have cloned and characterized eight *H. armigera* CTLs, of which CTL1 has been characterized to play a role in bacterial agglutination [37,38]. Another member CTL7, apart from having a function similar to CTL1, can also enhance cellular encapsulation and melanization [15,16]. Our in-depth immunotranscriptomic studies have identified a total of twenty-four CTLs (except CTL1 and 8), 18 of which are novel transcripts, much more than the PGRP and βGRP family size, thus significantly expanding our knowledge about CTL gene family in this devastating insect pest. In terms of domain structure (Figure 5A, in Additional file 6: Table S5), thirteen (CTL2-7, 11–14, 16, 25, and 26) had two tandem carbohydrate recognition domains (CRDs), while eleven (CTL15, 17–24) possessed only one. In addition to one CRD domain, CTL9 and 10 had three sushi and eleven sushi domains, respectively. Thirteen CTLs formed an independent clade in the phylogenetic tree, suggesting a possible gene expansion of this family in *H. armigera* (Figure 5). The phylogenetic analysis indicates that CTLs containing two CRDs are in the same group, suggesting a lepidopteran specific family expansion. *H. armigera* CTL2, 6, and 25 are orthologous to *M. sexta* immulectin-2, which agglutinated *E. coli* and might anchor the PPO activation reaction in the vicinity of microbial cells [39,40]. Along with this group, HaCTL7, 11, 26 were clustered together with MsIML-3 and MsIML4 in a bigger clade. HaCTL21, 22 and 23 formed three distinct clades along with the respective CTLs from *A. mellifera*, *T. castaneum*, *D. melanogaster*, *A. aegypti*, *B. mori*, and *Acyrthosiphon pisum.* These three clades are in turn part of a single branch in the phylogenetic tree which is distant from other major branches, and are characterized as galactose binding CTLs (CTLGAs) with QPD signature sequence. However, we could not find *H. armigera* homologue of *A. gambiae* CTL4 and CTLMA2, which prevented *Plasmodium* from melanization [41]. Our findings imply that *H. armigera* CTLs are much more diversified than other insect groups and may arise from gene duplications catering to specific pathogen interactions. Apart from CTLs, another class of lectins-Galectins that were found in diverse groups of insects and known to bind to β-galactoside were also essential for pathogen recognition [42]. *H. armigera* immunotranscriptome revealed three galectins, displaying 1 to 1 orthologous relationship with *B. mori* galectins (Figure 5B).

Some thioester-containing proteins (TEPs), belonging to C3/α-2 macroglobulin superfamily, harbor an intrachain β-cysteinyl-γ-glutamyl thioester bond, which is used for self- or non-self-identification [43]. α2-macroglobulin is a broad-spectrum protease inhibitor using a trapping mechanism through its thioester linkage. During the immune responses, complement factors C3, C4, and C5 bind to self/non-self surfaces via the same bond. In mosquitos, TEP family presents extensive gene duplication, with 15 genes in *A. gambiae*, 8 in *A. aegypti*, and 10 in *C. pipiens* [44]. In *A. gambiae*, TEP1 played the key role in blocking *Plasmodium* oocyst proliferation in the midgut [45]. *Helicoverpa,* however, has a relatively small TEP family with only four members which are homologous to *B. mori* TEPs (Additional file 1: Figure S6, in Additional file 6: Table S5). Since *H. armigera* TEP4 contains the signature motif GCGEQ, it is probably involved in immune surveillance against pathogens or parasites.

Based on sequence homology search, we have also identified other *H. armigera* PRRs encoded by 10 *scavenger receptors class B* (ScR-Bs), 1 *hemocytin*, 1 *hemolin*, 1 *DSCAM*, 1 *Draper*, 1 *RhoL*, 1 *Rap1*, 1 *enabled*, 1 *Fascin*, 1 *Scar*, 1 *Dizzy*, 1 *TM9SF4*, 1 *Integrin*, 1 *Noduler*, and 4 *Eater*, which are involved in phagocytosis, encapsulation, and various other cellular immune responses (in Additional file 6: Table S5) [46,47]. The phylogenetic analysis of the ScR-Bs (in Additional file 1: Figure S7) demonstrated that *H. armigera* and *B. mori* ScR-Bs were most likely to form the orthologous pairs. The *H. armigera* ScR-B9 might function in phagocytosis of the apoptotic bodies, since it is orthologous to *D. melanogaster* Croquemort (CG4280). The presence of a large number of PRR genes in the immunotranscriptome of *H. armigera*, indicated their involvement in the immune response to the infection of *B. bassiana* and *E. cloacae*.

### Genes involved in melanization and extracellular signal modulation

Upon PRR binding, the invading microbes may lead to the PPO activation. As key enzymes in the melanization reaction, the copper-containing phenoloxidases are activated from its inactive zymogens by specific proteolytic cleavage [28]. Active phenoloxidases catalyze the conversion of monophenols to *o*-diphenols and further oxidation of *o*-diphenols to quinones, which then polymerize to form melanin that entraps and kills microbial pathogens and parasites [8,48]. Except for mosquitoes, most insects had one to three PPOs [49]. Similar to other lepidopteran species, two PPO transcripts were identified in the *H. armigera* transcriptome. The *H. armigera* PPO1 and PPO2 share 80.3% and 79% of identity in amino acid sequence to the *B. mori* PPO1 and PPO2 (in Additional file 6: Table S5), respectively. The phylogenetic analysis establishes the orthologous groups of HaPPO1-BmPPO1-MsPPO1 and HaPPO2-BmPPO2-MsPPO2 (in Additional file 1: Figure S8).

Serine proteases (SPs) and their non-catalytic homologs (SPHs) constitute one of the largest protein family in insects that are functionally involved in various physiological processes such as digestion, development, hemolymph clotting, and immune responses [50]. Hemolymph SP cascades, comprised of multiple clip-domain SPs (cSPs), are essential for proteolytic activation of PPO and the processing of Toll pathway ligand, spätzle, which will be discussed in detail in the next section. In total, we have identified 46 SPs and 7 SPHs in *H. armigera* transcriptome (in Additional file 8: Table S7). Among them, 41 have complete sequences containing entire open reading frames (ORFs). Apart from these cSPs, five other SPs (SP41-44, 47) were shown to contain the structural modules important for protein-protein interactions, such as LDLA, Sushi, and SRCR. Detailed analysis of SPs revealed the orthology among *H. armigera* SP42, *M. sexta* HP14 and *D. melanogaster* modular SP. The respective SPs from *M. sexta* and *D. melanogaster* apparently are the first key enzymes triggering PPO activation and Spätzle processing [51,52]. Thus we postulated a similarly important role for *H. armigera* SP42, which needs further experimental verification.

**Figure 6:**
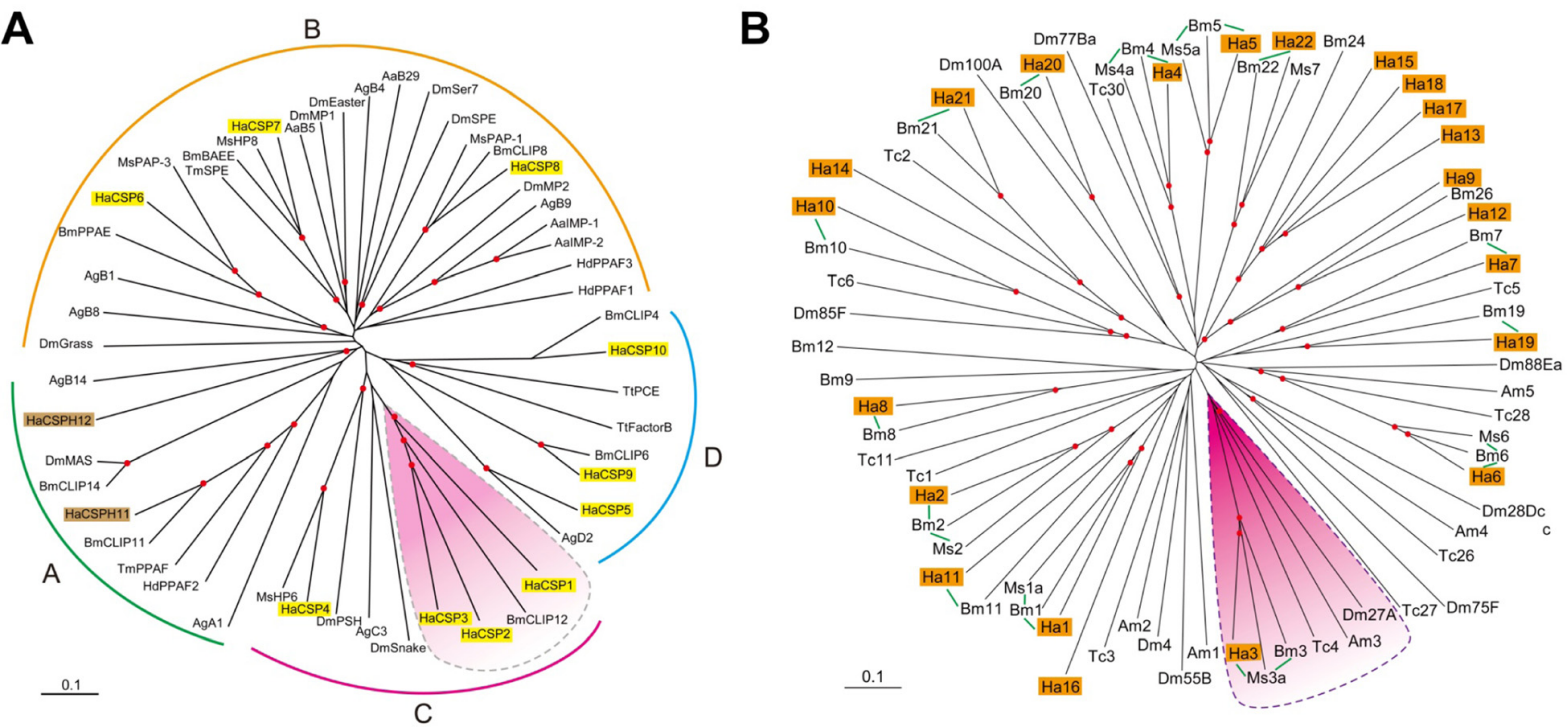
The clip-domain serine protease-related proteins (CLIPs) and serpins of *H. armigera*. **(A)** Phylogenetic analysis of CLIPs. The amino acid sequences of 12 *H. armigera* (Ha), 9 *D. melanogaster* (Dm), 8 *B. mori* (Bm), 3 *M. sexta* (Ms), 3 *H. diomphalia* (Hd), 4 *A. aegypti* (Aa), and 8 *A. gambiae* (Ag) CLIPs are compared and divided into four groups (A ~ D) based on sequence similarity. Scale bar, 0.1 substitutions per site. **(B)** Phylogenetic analysis of serpins. The amino acid sequences of 22 Ha, 11 Tc, 9 Dm, 18 Bm, 6 Ms, and 5 Am serpins are examined. The clade showing expansion compared with *B. mori* is shaded yellow. Scale bar, 0.1 substitutions per site. The red dots at nodes denote bootstrap values greater than 800 from 1,000 trials.

While each cSP harbors one or two regulatory modules at its amino terminus, clip-domain SPHs (cSPHs), although lacking catalytic serine residue, act as cofactors for cSP mediated reactions. In *Helicoverpa* transcriptome, 10 cSPs (cSP1-10) and 2 cSPHs (cSPH11-12) were identified (in Additional file 6: Table S5). This number is more or less similar to *B. mori* (18) but much lower than that of *D. melanogaster* (57) [31,50], partly due to the lack of the corresponding genome information. Multiple sequence alignment of the ten cSP catalytic domains suggests that they all have a trypsin-like specificity, based on residues predicted to form the primary substrate binding site (DGG) (in Additional file 1: Figure S9, in Additional file 8: Table S7). *H. armigera* cSPs and cSPHs are divided into four subfamilies based on their evolutionary relatedness (Figure 6A, in Additional file 6: Table S5). With similar SP subfamilies in other insects as well, these four groups of SP-related genes might represent lineages derived from ancient evolutionary events [53,54]. CLIPA is composed of cSPHs only. *H. armigera* cSPH11 of CLIPA is orthologous to *T. molitor* PPAF and *Holotrichia diomphalia* PPAF2 and based on this orthology a putative function of cSPH11 as a cofactor of PPO can be predicted [55,56]. Similarly, in CLIPB subfamily, cSP6 and cSP8 are orthologous to *M. sexta* PAP3 and PAP1 with 64.3% and 69.7% identity in amino acid sequences, respectively and may function as PPO activating protease [57,58]. Structurally, cSP6 harbors dual clip domains at its N-terminus, while cSP8 only has one. cSP8 and MsPAP1 were clustered together into a group which is close to but distinct from a clade comprising of DmMP2, AaIMP1, AaIMP2 and AgCLIPB9 in phylogenetic analysis. On the other hand, cSP6 was clustered together with *Ms*PAP3 and *Bm*PPAE into a clade distant and distinct from the above two clades. Likewise, *H. armigera* cSP7, belonging to yet another independent clade within CLIPB subfamily, is orthologous to MsHP8, BmBAEE and TmSPE, suggesting its involvement in spätzle processing and Toll pathway activation [12,59]. CLIPC includes four cSPs, of which cSP4 forms an independent branch as that of *M. sexta* HP6 and *D. melanogaster* Persephone. Since *M. sexta* HP6 and HP8 constituted a branch leading to the cleavage of Spätzle, *H. armigera* cSP4 and cSP7 are postulated to achieve the same function.

The PPO activation is delicately regulated by serpins. Serpins are a superfamily of proteins, which have around 350–400 amino acid residues and the conserved tertiary structure comprising nine α-helices and three β-sheets [60]. Most of them inhibit SP-mediated processes by forming covalent complexes with their cognate SPs. The P1 residue at an exposed reactive site loop (RSL) near the carboxyl terminus determines its inhibitory selectivity. Sequence similarity search of *H. armigera* unigenes yields 22 serpin transcripts, most of which have putative orthologs in *B. mori* (Figure 6B, in Additional file 6: Table S5). Of these, sixteen serpins have a predicted signal peptide for secretion, while the rest (Serpin2, 4, 10, 12, 16, 21) are assumed to be cytosolic (in Additional file 6: Table S5). *H. armigera* Serpin1-6 are highly similar to their *M. sexta* and *B. mori* orthologs, suggestive of their essential roles in regulating innate immunity [28]. One interesting feature of *M. sexta* and *B. mori* Serpin1 and 2 is the mutual exclusive use of alternative exons to generate RSL variants on the same framework. While initial analysis suggests the same mechanism in *H. armigera* (data not shown), a detailed study of Serpin1 and 2 gene sequences will reveal the scope of this phenomenon. 1:1 orthologous relationship between *H. armigera*, *B. mori*, and *M. sexta* Serpins3, 4 and 5 allowed us to make predictions about the putative functions of these HaSerpins as summarized below [61,62]. *D. melanogaster* Spn27A, *M. sexta* serpin-3 and *B. mori* Serpin3 are negative regulators of the melanization cascade. In the phylogenetic tree, *H. armigera* Serpin3 was located in the same clade with them and also contained the conserved P2-P2′ sequence (NK-FG) (Figure 6, in Additional file 1: Figure S10). Serpin3 may negatively regulate melanization and the activation of Spätzle, while Serpin4 and 5 might inhibit hemolymph cSPs regulating immune response in general (Figure 6, in Additional file 1: Figure S10). We predict their proteolytic cleavage site based on the multiple sequence alignment of the hypervariable RSLs of the *H. armigera* serpins (in Additional file 1: Figure S10). Serpin10 and 14 lack a conserved carboxyl-terminus and may not fold properly with a long insert separating the partial serpin domain. Serpin1, 3 ~ 6, 8, 9, 13, 21, and 22 (with Arg or Lys located at the predicted P1 position) may inhibit trypsin-like enzymes, while serpin-2, 7, 11, 12, and 17 ~ 20 (with Phe, Tyr, or Leu located at the putative P1 site) are anticipated to inhibit chymotrypsin-like SPs. Only three serpins, Serpin12, 15, and 20, contain Val and Met at the P1 position to control elastase-like enzymes. These predicted inhibitory activities and selectivity features surely need further experimental data to validate.

### The immune signaling pathways and effector gene families

**Figure 7:**
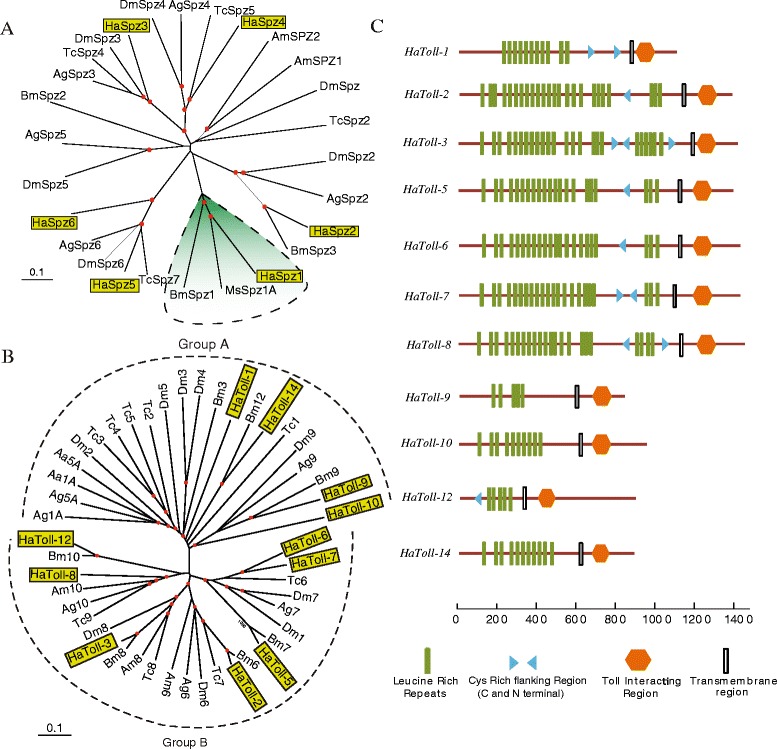
The spätzles and Toll-like receptors of *H. armigera*. **(A)** Phylogenetic analysis of spätzle homologs. The amino acid sequences of 6 *H. armigera* (Ha), 4 *T. castaneum* (Tc), 6 *D. melanogaster* (Dm), 3 *B. mori* (Bm), 1 *M. sexta* (Ms), 5 *A. gambiae* (Ag), and 2 *A. mellifera* (Am) spätzles are compared. The red dots at nodes denote bootstrap values greater than 800 from 1,000 trials. Scale bar, 0.1 substitutions per site. **(B)** Phylogenetic analysis of Toll-like receptors from seven insect species. The amino acid sequences of 11 Ha, 9 Tc, 9 Dm, 7 Bm, 2 Aa, 6 Ag and 3 Am Toll-like receptors are examined. Scale bar, 0.1 substitutions per site. **(C)** Schematic representations of the domain structure of *H. armigera* Tolls. Lengths of the amino acid sequences are indicated.

**Figure 8:**
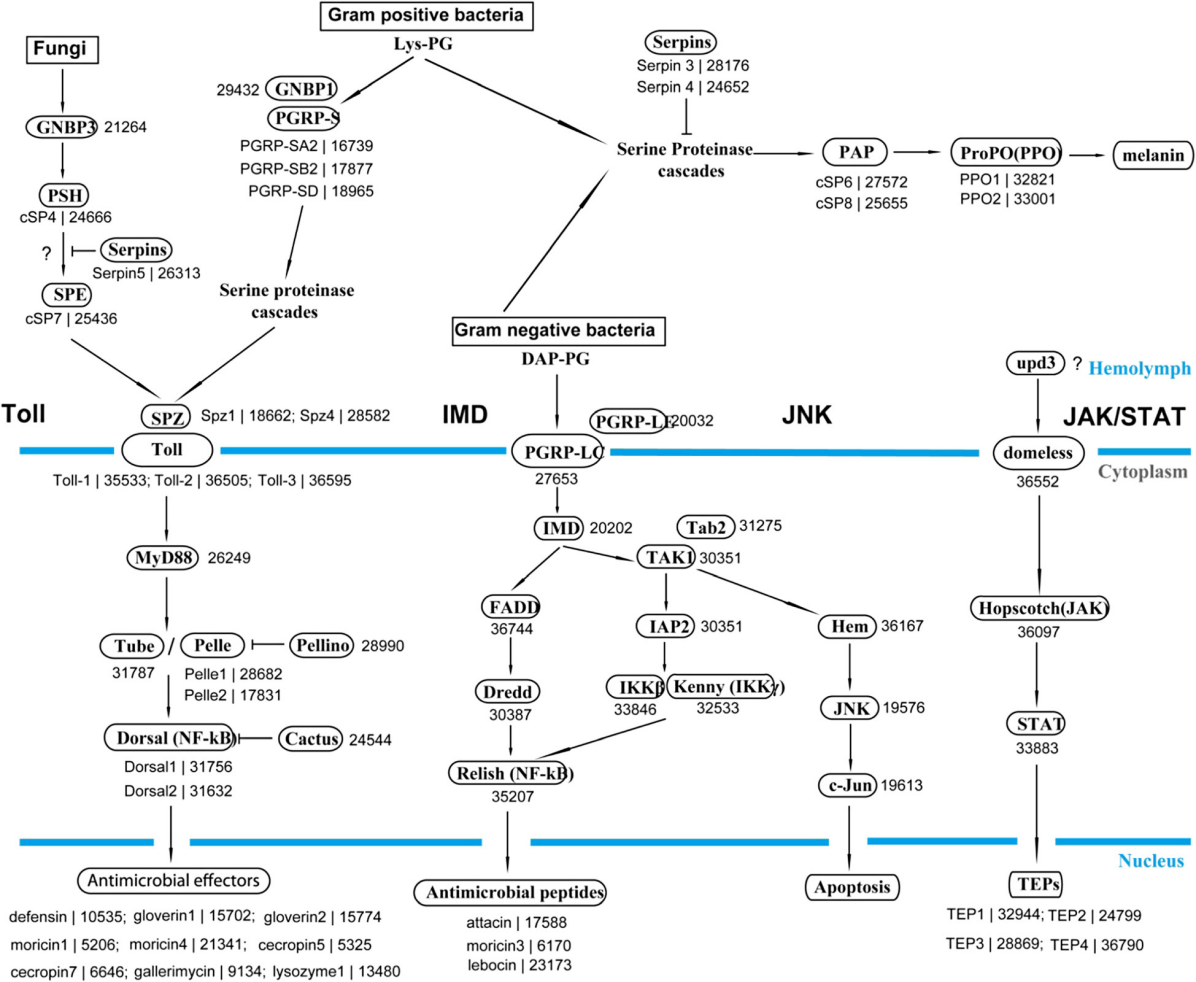
Putative *H. armigera* members for major immune signaling pathways. Illustrated were Toll, IMD, JNK, JAK-STAT, and melanization pathways that were revealed in *D. melanogaster* and *M. sexta*. Below names were candidate *H. armigera* members predicted based on sequence similarity. *H. armigera* gene names are also followed by corresponding Unigene numbers.

After recognizing the surface molecules of invading pathogens, recruited hemolymph immune factors would trigger signal transduction pathways and induce the production of effector molecules. Similar to melanization, the Toll pathway is also involved in the antifungal and antibacterial immunity. Cleavage activated Spätzle by *Drosophila* SPE (orthologous to *M. sexta* HP8 and *H. armigera* cSP7) acts as the ligand for this pathway, binding to Toll receptors and triggering its dimerization between leucine-rich repeats [6,7,63]. Six spätzle (Spz) transcripts are present in the *H. armigera* transcriptome (Figure 7A, in Additional file 6: Table S5). The phylogenetic tree indicates *H. armigera* Spz2 ~ 4 are 1:1 orthologs of *D. melanogaster* Spz2 ~ 5. *H. armigera* Spz1 contained 9 Cys residues at the same position to, perhaps form one interchain and four intrachain disulfide bonds. *H. armigera* cSP7, a trypsin-like enzyme, may cleave after R^119^ to activate the Spätzle-1. Orthologous to *B. mori* and *M. sexta* Spz1s with 41.9% and 53.5% identity, respectively and are grouped together in a lepidoptera specific branch in the phylogenetic analysis [64]. Toll like receptors have also undergone significant diversification, with a total of 11 members in *H. armigera* transcriptome (Figure 7B, in Additional file 6: Table S5). They contain the entire ORFs encoding extracellular Leu-rich regions, transmembrane region, and cytoplasmic TIR domains (Figure 7C). The phylogenetic analysis supported the following orthologous relationship: *Dm*9-*Ag*9-*Ha*9-*Bm*9. Like *D. melanogaster* Toll9 [65], *H. armigera* Toll-9, may participate in the induced synthesis of antifungal proteins. *H. armigera* Toll-6 and Toll-7 may also play roles in immune signaling, as they both are in the same clade along with *D. melanogaster* Toll1 and Toll7. Although the Toll pathway ligands and receptors have experienced major family expansions, the corresponding intracellular components, such as MyD88, Tollip, Tube, Pelle, Pellino and Cactus, are mostly conserved in all the studied insects including *H. armigera* (in Additional file 6: Table S5). Our results suggested that these components formed a multimeric protein complex in *H. armigera*, which further phosphorylated HaCactus resulting in the production of AMPs. The immune molecules involved in the anti-fungal and -bacteria pathways were conserved in the *H. armigera* (Figure 8).

The insect IMD pathway, analogous to the mammalian TNF signaling pathway, is critical for combating Gram-negative bacteria [7]. IMD protein itself is similar to mammalian TNF receptor as both contain a homologous death domain. Activation of IMD leads to the proteolytic cleavage of a Rel family transcription activator, Relish. The released active domain of Relish is then translocated into the nucleus to exert its function [6]. The IMD pathway components, including IMD, Dredd, FADD, TAK1, Tab2, IKK-β, IKK-γ and Relish were all identified in the *H. armigera* transcriptome strongly suggesting the conservation of IMD mediated immunity in cotton bollworm (in Additional file 6: Table S5). Additionally, two members of IAP-2 were also identified. In *D. melanogaster*, IAP-2 acts as a modulator of IMD pathway aiding the nuclear localization of Relish [66].Besides Toll and IMD pathways, JNK and JAK/STAT pathways are also implicated to play a role in insect immunity. Activated by TAK1 kinase, JNK pathways function through transcription factor Relish resulting in production of AMPs in both *A. gambiae* and *D. melanogaster* [67]. One copy of *JNK*, *Hem*, *Jun* and *Fos* genes of the JNK pathway were all identified in *H. armigera* transcriptome (in Additional file 6: Table S5). In *Drosophila,* JAK/STAT pathway controls the transcription of some immune molecules including TEPs, promotes phagocytosis and participates in antiviral response [68]. Based on homology to *D. melanogaster* JAK/STAT pathway members, cytokine receptor *Domeless*, *Hopscotch*, and *STAT* were present as single copy identified in *H. armigera*, indicating the conservation of this pathway (in Additional file 6: Table S5). The negative regulators of JAK/STAT pathway, *SOCS* and *PIAS*, were found in the transcriptome as well. Taken together, all these four pathways-Toll, IMD, JNK and JAK/STAT, coordinates in relaying the ‘danger’ signal ultimately stimulating the production of immune effector molecules (Figure 8).

Most AMPs are low molecular weight (<30 kDa) proteins forming amphiphilic α-helix, hairpin-like β-sheet, or structures stabilized by disulfide bonds [69]. Induction of AMPs in fat body and hemocytes is thoroughly studied in lepidopteran insects including *M. sexta* and *B. mori* [28,31]. Analysis of the *H. armigera* transcriptome revealed unigenes homologous to AMPs, including 4 lysozymes, 5 cecropins, 4 moricin, 3 gloverins, 1 defensin, 1 gallerimycin, and 1 lebocin (in Additional file 6: Table S5). The number of annotated AMPs is much lower than that of *B. mori* [31] and *M. sexta* [32], probably due to the lack of genome information. While cecropins and defensins are found in various insects, moricins and gloverins are only reported to be present in Lepidoptera. Furthermore, four putative lysozyme transcripts are present in *H. armigera* transcriptome (in Additional file 1: Figure S11). Lysozymes are 14–16 kDa enzymes that hydrolyze peptidoglycans in bacterial cell wall [70]. All four *H. armigera* lysozymes belong to the c-type lysozymes with four disulfide bridges, and lysozymes 1–3 contain the conserved active site including the Asp and Glu residues. Reactive oxygen and nitrogen species (ROS and RNS) played an important role in killing microbes and parasites [71]. However, the overproduction of free radicals can be toxic to the host cell, therefore a tight regulation of ROS/RNS concentration is required. Superoxide dismutase (SOD), glutathione S-transferase (GST), or peroxiredoxin converts ROS/RNS to less toxic product. Here, we have annotated some of genes, including 5 *GSTs*, 3 *SODs*, and 6 *peroxiredoxin* transcripts (in Additional file 6: Table S5), indicating that free radicals produced by ROS and RNS are likely to be components of the defense system in *H. armigera*. Further experiments are needed to verify suggested role of the immune signaling pathways and effectors in *H. armigera* as described above.

### Expression profile of immunity-related genes in the fat body after fungal and bacterial infections

**Figure 9:**
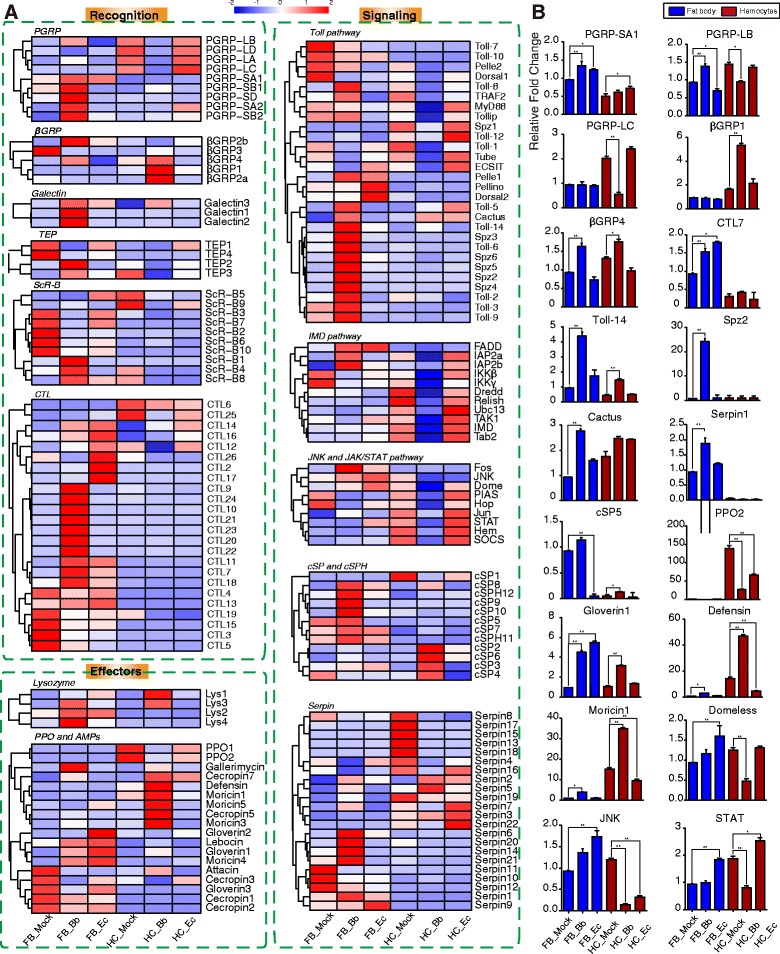
Expression patterns of the *H. armigera* immunity-related genes. **(A)** Cluster analysis. Expression profiles of immunity-related genes data are organized into three groups: recognition, signaling, and effectors. Fat bodies and hemocytes are extracted from *H. armigera* larvae infected by Bb 48 post infection and Ec 6 post infection respectively. Six datasets were included: FB_Mock, FB_Bb, FB_Ec, HC_Mock, HC_Bb, HC_Ec. Gene families and functional pathways (Toll, IMD) are categorized within the group. Gene names are shown on the right side. **(B)** Quantitative real-time PCR analysis of the *H. armigera* immunity-related gene expression in hemocytes and fat body of the fifth instar larvae after *B. bassiana* (48 h) and *E. cloacae* (6 h) injection. *H. armigera* ribosomal protein S3 (*rps3*) was used as an internal standard to normalize the templates. The relative mRNA levels are represented as the mean ± S.D. (*n* = 3). *, p < 0.05; **, p < 0.01. FB_Mock, control fat body; FB_Bb, fungus-induced fat body; FB_Ec, bacterium-induced fat body; HC_Mock, control hemocytes; HC_Bb, fungus-induced hemocytes; HC_Ec, bacterium-induced hemocytes.

To verify the FPKM value changes between control and treatment groups, we performed quantitative real-time PCR analysis of the expression profiles of 18 genes in fat body and hemocytes from *B. bassiana*-, *E cloacae-* and buffer-injected *H. armigera* larvae. The results, consistent with the deep RNA-seq data, provide an overview of tissue-specific gene expression patterns of *H. armigera* larvae in response to *B. bassiana* infection (Figure 9, and in Additional file 9: Table S8). This suggests the expression data of other genes from the RNA-seq data analysis are also reliable.

Humoral defense proteins are mainly produced and secreted by fat body, an insect tissue analogous to liver and adipose tissue of mammals in terms of immunity and metabolism. For PRR genes, RNA-seq based transcriptome analysis indicated that short PGRPs (PGRP-SA1, -SA2, -SB1, -SB2, and -SD) were induced in fat body after the fungal challenge. Similarly, transcription of ScR-B1, B4, B8, TEP2, TEP3, CTL9 ~ 11, 18, 20 ~ 24 and galectins is up-regulated. This change is not limited to PRRs, some signal transducers and regulators are also induced. Cluster analysis of expression patterns revealed that cSP5, 8 ~ 10, cSPH11, 12, Serpin6, 14, 20, 21 mRNAs became much more abundant in fat body after the fungal challenge whereas higher levels of cSP2 ~ 4 and cSP6 transcripts were detected in hemocytes. The pronounced up-regulation of Toll pathway genes (*e.g*. Spz2, Pelle, Toll-8, Toll-9, Toll-14) occurred in fat body elicited by *B. bassiana*, suggesting their involvement in antifungal immunity. The gloverin1, lysozyme2, and lysozyme4 mRNA levels increased most dramatically to defend the fungal infection. Notably, most of these genes did not change sharply at transcript level in the other experiment conditions, indicating that fat body plays a major role in the antifungal response.

Bacterial challenge also resulted in the activation of a broad spectrum of immunity-related genes. These include PRRs such as CTLs (CTL2, 12, 14, 16, 17, and 26), signaling modulators (cSP3, Serpin1, 4, and 9), and effector molecules (gloverin1, gloverin2, Lebocin, and Moricin4). The mRNA abundances of FADD, Fos, JNK significantly increased in fat body elicited by *E. cloacae*, as evidenced by 12 genes in cluster 4, which were significantly up-regulated (fold change > 2, *P* < 0.001) in response to such bacterial infection. This number was apparently lower than that elicited by fungi, which is 24 (cluster 3, Figure 1C). Although more immunity-related genes were elicited by fungi (67) than bacteria (42), there is an overlap between them, including up-regulated cSP8, lysozyme3, gloverin1 and hemolin, and down-regulated βGRP3 (in Additional file 1: Figure S12).

### Expression profile of immunity-related genes in hemocytes in response to fungal and bacterial challenges

Hemocyte mediated defense reactions such as phagocytosis and encapsulation also depend on the recognition of foreign pathogens to trigger downstream immune signaling pathways. The expression profiles of immunity-related genes in hemocytes elicited by the pathogens were highly different from those in fat body. To better understand the complexity of responses of hemocytes against pathogens, the expression profile of immunity-related genes elicited by fungi and bacteria was compared. Gene cohorts of cluster 1 include 21 such genes induced in *B. bassiana* induced hemocytes, including βGRP1, βGRP2, hemolin, cSP2, cSP6, Serpin2, Serpin5, lysozyme1, lysozyme3 and so on. Immune activation by *E. cloacae* is also noticeable as demonstrated by induction of PRRs (PGRP-SA2, TEP1) and immune modulators (MyD88, Toll-5, Cactus, Toll-12, Serpin3, 11, 21).

Thirty-three immunity-related genes in cluster 2 were significantly suppressed in *B. bassiana* elicited hemocytes. RNA-seq based transcriptome analysis indicated that long PGRPs (PGRP-LA, -LB, -LC, -LD) were strongly suppressed by *B. bassiana* but induced by *E. cloacae*. Quantitative real-time PCR also revealed that the βGRP1 mRNA level dramatically increased in hemocytes elicited by *B. bassiana*. In contrast, CTL mRNA levels did not change significantly in hemocytes. The transcript levels of PPOs were high in hemocytes, but significantly decreased in response to fungal challenge. Likewise, components of the IMD pathway (FADD, IAP2a, 2b, IKKβ, IKKγ, Dredd, Relish, UBc13, TAK1, IMD, Tab2), JNK and JAK-STAT pathways (Domeless, PIAS, Hopscotch, STAT, Fos, JNK, Hem, SOCS) were all dramatically suppressed in hemocytes in response to the fungal infection but moderately induced by bacterial infection, as validated by the quantitative real-time PCR assay of Domeless, JAK, and STAT transcripts. This indicated that signal transduction in hemocytes responds differently to bacterial and fungal infection. More immunity-related genes were regulated by fungi (60) than bacteria (18) in hemocytes, and most of them tended to be suppressed (in Additional file 1: Figure S12).

Although fat body and hemocytes both are important organs involved in the immune response, our transcriptome analysis revealed that more immunity-related genes are induced in fat body (clusters 3 and 4) than hemocytes (cluster 1). The expression of immune recognition and modulation molecules was mainly controlled in fat body, while genes involved in signal transduction, such as JAK-STAT and IMD, were regulated more drastically in hemocytes (Figure 9). In Toll pathway, hemocytes regulated Toll-1, Toll-5, Toll-12, Tollip, Cactus, and MyD88, but more other genes were regulatedin fat body. Additionally, quantitative real-time PCR indicated that the transcription of putative PGRP-SA1, PGRP-LB, Toll-14 and Spz2 genes were elevated in fat body upon *B. bassiana* infection, while the mRNA levels of defensin, moricin and gloverin1 were up-regulated in hemocytes (Figure 9), which is consistent with the RNA-seq results.

## Conclusions

While many immunological studies are focused on *B. mori* and *M. sexta*, *H. armigera*, as a notorious lepidopteran pest affecting world agriculture, has not yet been systematically investigated on aspects of immunity until now. In order to develop and apply better pest control methods, we used high throughput sequencing to analyze the transcriptome of hemocytes and fat body in response to bacterial and fungal infection. The 233 immunity-related transcripts discovered are involved in recognition, signal transduction and modulation, and execution mechanisms. This number is certainly going to increase when its genomic information is available, based on the information acquired from this study, we can conclude that the repertoire of immunity-related genes is well conserved in *H. armigera*. Moreover, we find that these genes displayed distinct mRNA profiles in response to *B. bassiana* and *E. cloacae* infection. Their products may constitute an integrated pathway of antifungal immunity, which provide valuable clues for further functional studies in *H. armigera* responding to *B. bassiana*, and pave the way for using *H. armigera* as a model system to study fungal-host interaction [72]. We have achieved another goal of using the transcriptome dataset to reveal tissue-specific gene expression patterns. The global patterns of hemocytes and fat body remarkably differ from each other after infection by the two microbes. Furthermore, it is demonstrated that not only immunity-related genes but also metabolism genes are under the regulation of pathogen infection.

Taken together, this study provided a global view of host defense gene expression profiles in response to bacterial and fungal challenges in a non-model insect. The results could benefit future in-depth study on the role of candidate genes involved in anti-fungal and -bacterial immunity, thereby helping improve the understanding of host-pathogen interactions and evolutionary history of immunogenetics from insects to mammals.

## Methods

### Insect rearing, bacterium and fungus culture

A colony of *H. armigera* was obtained from Dr. Qi-Lian Qin at Institute of Zoology, Chinese Academy of Sciences, and maintained in the laboratory. Larvae were reared on an artificial diet at 28 ± 1°C under a photoperiod of 14:10 (l: d) and 70% relative humidity. Male and female pupae were placed in separate glass dishes under the same conditions for eclosion. Adults were fed with 10% honey solution. The colony of *H. armigera* in the laboratory was maintained for over five generations before experiments. *E. cloacae* strain (strain no. 1.2022) was obtained from China General Microbiological Culture Collection Center. The bacteria at stationary phase in LB broth (A_600_ ≈ 2.0) were diluted four times (A_600_ ≈ 0.5) and used for septic injury. *B. bassiana* strain ARSEF 2860, was cultured on potato dextrose agar (PDA) plates at 25°C and 80% humidity [73].

### Septic injury and RNA sample preparation

At day 2 after molting, approximately 120 fifth instar larvae from the same batch were divided into three groups. Each larva in the treated groups was inoculated in hemocoel with 2 μl of the *B. bassiana* suspension (1 × 10^7^ conidia/ml), *E. cloacae* suspension (A_600_ ≈ 0.5), or sterile PBS using a microinjector. Then all the larvae were kept under the same rearing conditions. The samples were collected from *E. cloacae* infected larvae at 6 h and *B. bassiana* infected larvae at 48 h, respectively, at which time larvae started to die. Hemolymph samples were individually collected into ice-cold PBS containing 0.1% 1-phenyl-2-thiourea. Suspensions were pooled in a 1.5 ml Eppendorf tubes and centrifuged at 500 *g* for 5 min at 4°C to collect hemocytes. Fat body tissues were removed from larvae under a dissection microscope. Control samples from the PBS-injected larvae were prepared at 6 h and 48 h in the same way, and RNA was combined in equal amount.

Samples of hemocytes and fat body were homogenized using a motor-driven pellet pestle mixer (Kontes, USA) and lysed in 1.0 ml of TRI Reagent (Sigma, USA). Total RNA was extracted using RNeasy Mini Kit (Qiagen, USA) according to the manufacturer’s instructions. RNA concentrations were determined on a Nanodrop ND-2000 spectrophotometer (NanoDrop products, USA). RNA integrity was verified on Agilent 2100 BioAnalyzer (Agilent, USA).

### Survival analysis

Day 2 fifth instar *H. armigera* larvae (24 in each group) were individually challenged with *B. bassiana* or *E. cloacae*. Insects in the treatment groups and control group of buffer injection were maintained in individual containers and fed on the standard diet. Dead larvae were counted at different time intervals for calculation of mortality rate. The survival curves were compared using the Kaplan-Meier and Cox’s proportional hazards model to assess variables that affected survival [74]. The threshold of p value was adjusted by the Bonferroni correction. GraphPad software was used in all statistical analysis. The experiment was repeated three times.

### Library construction and Illumina sequencing

Ten μg of total RNA from each treatment or control group was used to enrich poly(A) mRNA using oligo(dT) magnetic beads (Invitrogen, USA). In the next step, paired-end RNA-seq libraries were prepared by following the Illumina’s library construction protocol. The challenged and control libraries were sequenced on Illumina HiSeq 2000 platform (Illumina, USA) in the Beijing Institutes of Biological Sciences (Chinese Academy of Sciences). FASTQ files of raw-reads were produced and sorted out by barcodes for further analysis.

### Assembly of transcriptomes and functional classification

Prior to assembly, 2 × 100 bp paired-end raw reads from each cDNA library were processed to remove adaptors, low quality sequences (Q < 20), and reads contaminated with microbes using Trimmomatic package [75]. The FastQC package was used to verify the quality of resulting trimmed and filtered reads. The clean reads were *de novo* assembled to produce contigs using Trinity, the short reads assembling program [19,20] using default parameters. One set of transcript contigs was assembled for each library. To increase the transcriptome coverage, second instar larvae were injected with the bacteria, fungi or PBS (Xiong and Zou, unpublished data). Whole body mRNA samples were prepared and sequenced in the same way. Clean reads from the nine samples were assembled into the tenth dataset, which had the most full-length CDSs and splicing variants. To reduce redundancy, CAP3 [76] was used to link overlapping transcripts into larger contigs. The final collection of transcripts was made up from consensus cluster sequences and singletons. The final collection of reference transcripts (or unigenes) was made up of consensus cluster sequences and singletons.

Non-coding RNAs were obtained using homologous search NONCODE [77]. For functional annotations, we searched all the assembled sequences against the NCBI non-redundant (NR) sequence database using BLASTX, with an E-value cut-off of 1e^−5^. An in-house Perl script was used to identify putative coding regions from the sequences, and translate them into amino acid sequences for further analysis. To determine enriched GO terms, significantly up- and down-regulated genes were subjected to gene ontology enrichment analysis through BLAST2GO against GO database [21,25]. Finally, KEGG analysis was performed to discover the enriched pathways and metabolic networks via online tool KAAS at the KEGG website [26]. In addition, GO annotations were performed for all unigenes with BLAST2GO program according to the GO association found by a BLASTX search against the NR database. For GO enrichment analysis, the corrected p value less than 0.05 were considered as significantly enriched in DETs. For KEGG pathway enrichment analysis, pathways with Q value less than 0.05 was regarded as statistically significant.

### Identification and hierarchical clustering of differentially expressed transcripts (DETs)

Transcripts abundance were quantified as follows: First, aligning the filtered raw paired-end reads from each of the six tissue-specific library to the corresponding non-redundant *H. armigera* unigenes using Bowtie [78]. Next, RSEM [79] package was applied for calculation of normalized gene expression value FPKM (an estimate of the number of sequence reads that are originated from a given transcript, accounting for the possibility that a single read could align to multiple transcripts). Subsequently, DETs between the control and treatment libraries were calculated based on the significance level (p value < 0.001) using DEGseq package in R environment [23]. These transcripts with expression difference more than 2 or less than 0.5 folds were considered as up- and down-regulated genes, respectively. Hierarchical clustering of DET was performed using heatmap.2 function within gplots package in R environment, where the *average* method was used to compute the hierarchical clustering and *Pearson* correlation method was adopted to calculate the distance between both rows and columns. For principal component analysis (PCA), PCs, PC variances, and PC scores were calculated using eigen function in R language [22].

### Comparative analysis of immunity-related genes from *H. armigera* transcriptome

The available immunity-related gene sequences from other model insect species were used as templates to search the *H. armigera* unigene sequence collection. The reference insect species include *D. melanogaster* [49], *A. gambiae* [53], *A. mellifera* [80], *B. mori* [31], and *T. castaneum* [27]. The potential candidates of *H. armigera* immunity-related genes were confirmed by searching the BLASTX algorithm against the Nr database using a cut-off E-value of 0.1. The deduced amino acid sequences were analyzed by Pfam (http://pfam.xfam.org/) and SMART (http://smart.embl-heidelberg.de/) to detect conserved domain structures required for specific functions. Signal peptide predictions were determined by SignalP4.0.

### Phylogenetic analyses

*H. armigera* sequences were aligned with their homologues from other representative insect species from holometabolous insect orders, including *D. melanogaster*, *A. gambiae*, *A. mellifera*, *T. castaneum*, and *B. mori*. The sequences were retrieved from NCBI, Immunodb, or Ensembl. Multiple sequence alignments were performed by CLUSTALX software with BLOSUM series of weight matrices [81]. Phylogenetic trees were constructed by the neighbor-joining method with statistical analysis by the bootstrap method, using 1000 repetitions [72].

### Quantitative real-time PCR analysis

To validate the expression profile from RNA-seq results, specific primers were designed to perform a quantitative real-time PCR analysis of selected immunity-related genes (in Additional file 9: Table S8) [11]. The annealing temperatures of the primers were controlled at about 62°C. 2 μg of total RNA was treated with DNase I (Invitrogen, USA) to remove contaminating genomic DNA, and then used for cDNA synthesis by M-MLV reverse transcriptase (Promega, USA). Ribosomal protein S3 was used as an internal standard to normalize cDNA templates. The quantitative real-time PCR was performed on a MX3000P system (Stratagene, USA) using SYBR green PCR Master Mix (Tiangen, China), according to the manufacturer’s instructions. The thermal cycling conditions were: 94°C, 5 s; 59°C, 20 s; 72°C, 20 s. Quantitative real-time PCR data were collected and exported to EXCEL for analysis.

### Sequence data submission

All the raw sequence data and the final *Helicoverpa* transcriptome described in the manuscript are available under the BioProject accession No. PRJNA264881. Illumina sequence reads have been deposited at NCBI SRA database under the following accession numbers (FB_Mock: Sample: SAMN03120571, Experiment: SRX735419, Reads: SRR1630741; FB_Bb: Sample: SAMN03144202, Experiment: SRX743611, Reads: SRR1630794; FB_Ec: Sample: SAMN03144203, Experiment: SRX743612, Reads: SRR1630796; HC_Mock: Sample: SAMN03144201, Experiment: SRX743613, Reads: SRR1630797; HC_Bb: Sample: SAMN03144199, Experiment: SRX743614, Reads: SRR1630798; HC_Ec: Sample: SAMN03144200, Experiment: SRX743615, Reads: SRR1630799). *H. armigera* unigenes are available under NCBI TSA accession number affiliated with the same BioProject.

## Additional files

Additional file 1: Figure S1. Morphological characteristics of microbial pathogens. (A) The entomopathogenic fungi were separated into conidia (blue arrowhead) and hyphae (red arrowhead). (B) The rod-shaped bacteria *E. cloacae* were colored light pink (red arrowhead) after Gram staining. **Figure S2.** Flowchart of the RNA-seq analysis. **Figure S3.** Length distribution of the *H. armigera* cDNA contigs and their comparison with homologs in other insect species. (A) Length distribution of the contigs assembled using different strategies. (B) Comparison of the initially obtained unigenes with homologous sequences in other insects. **Figure S4.** Identification of DETs in the *H. armigera* immunotranscriptome. (A) The MA-plot displays the DETs identified from different comparisons. *M* is the *Y*-axis representing the intensity ratio, and *A* is the *X*-axis representing the average intensity for each transcript. (B) Comparison of DEGs in hemocytes and fat body infected by *B. bassiana* and *E. cloacae*. **Figure S5.** Multiple sequence alignment of PGRP domains. **Figure S6.** Phylogenetic analysis of the thioester proteins in *H. armigera* and other insects. **Figure S7.** Phylogenetic analysis of the scavenger receptor Bs in *H. armigera* and other insects. **Figure S8.** Phylogenetic analysis of prophenoloxidases in *H. armigera* and other insects. **Figure S9.** Sequence alignment of the catalytic domains in SPs and SPHs. The green and yellow shades indicate the conserved residues. Determinants of the primary substrate-binding pocket are shade cyan. **Figure S10.** Reactive site loops of the *H. armigera* serpins. Gap positions are manually adjusted for better alignment. Predicted P1 residues are shown in bold and red. **Figure S11.** Phylogenetic analysis of the lysozymes in *H. armigera*. **Figure S12.** Venn diagram showing the unique and shared immunity-related DETs in fat body and hemocytes after the fungal and bacterial infection. The overlapping regions represent genes concomitantly regulated under the two experimental conditions. Additional file 2: Table S1. General features of the *de novo* assembled transcripts by Trinity. Additional file 3: Table S2. Putative long noncoding RNAs identified using NONCODE database. Additional file 4: Table S3. The gene ID, length, and predicted function of each unigene. Additional file 5: Table S4. Transcript levels of DETs present in at least two libraries. Additional file 6: Table S5. Immunity-related genes identified in the *H. armigera* transcriptome. Additional file 7: Table S6. Deduced amino acid sequences of putative immunity-related unigenes. All sequences were listed in FASTA format. Additional file 8: Table S7. Serine proteinases and their homologs in *H. armigera.*
Additional file 9: Table S8. Primers used for quantitative real-time PCR analysis.
