# Supplementary material for: High throughput profiling of the cotton bollworm Helicoverpa armigera immunotranscriptome during the fungal and bacterial infections
Source: BMC Genomics. 2015 Apr 18;16(1):321. doi: 10.1186/s12864-015-1509-1 (PMC4490664; doi:10.1186/s12864-015-1509-1)

Fig. S1

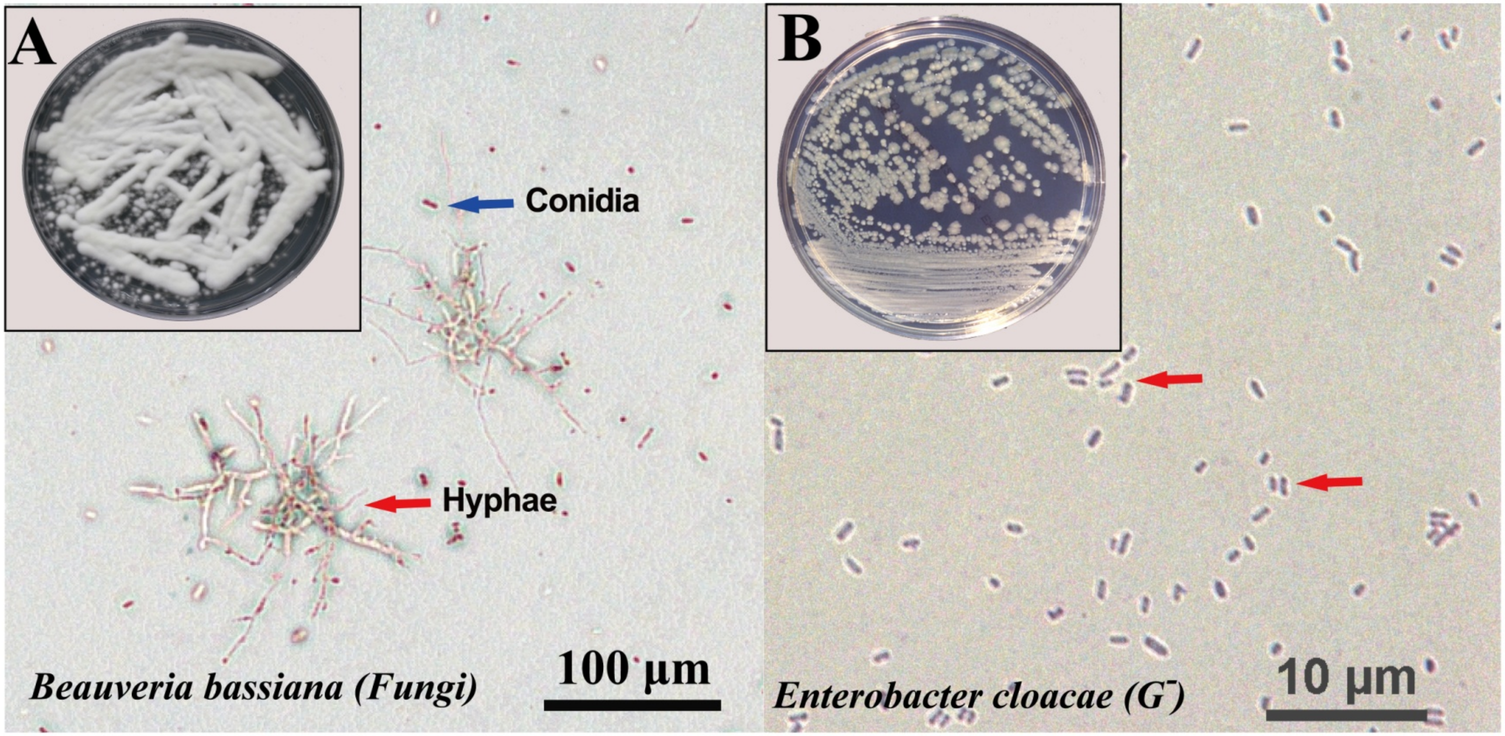
A
Conidia
Hyphae
Beauveria bassiana (Fungi)
100 µm
B
Enterobacter cloacae (G⁻)
10 µm

Fig. S2

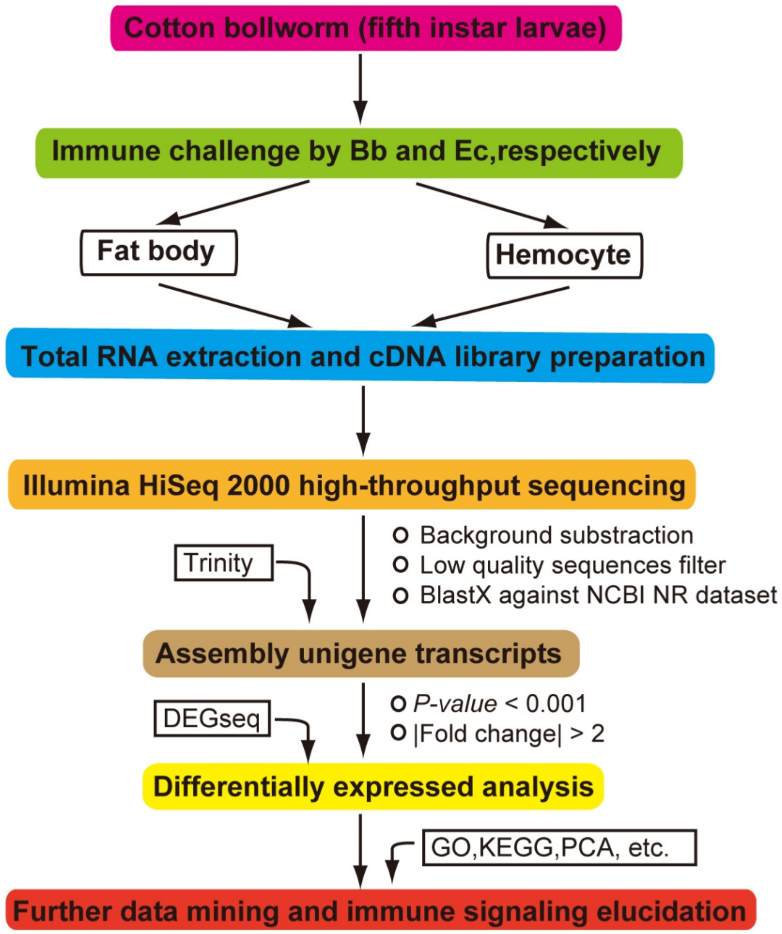
Cotton bollworm (fifth instar larvae)
Immune challenge by Bb and Ec,respectively
Fat body
Hemocyte
Total RNA extraction and cDNA library preparation
Illumina HiSeq 2000 high-throughput sequencing
Background substraction
Low quality sequences filter
BlastX against NCBI NR dataset
Trinity
Assembly unigene transcripts
DEGseq
P-value < 0.001
|Fold change| > 2
Differentially expressed analysis
GO,KEGG,PCA, etc.
Further data mining and immune signaling elucidation

Fig. S3

A

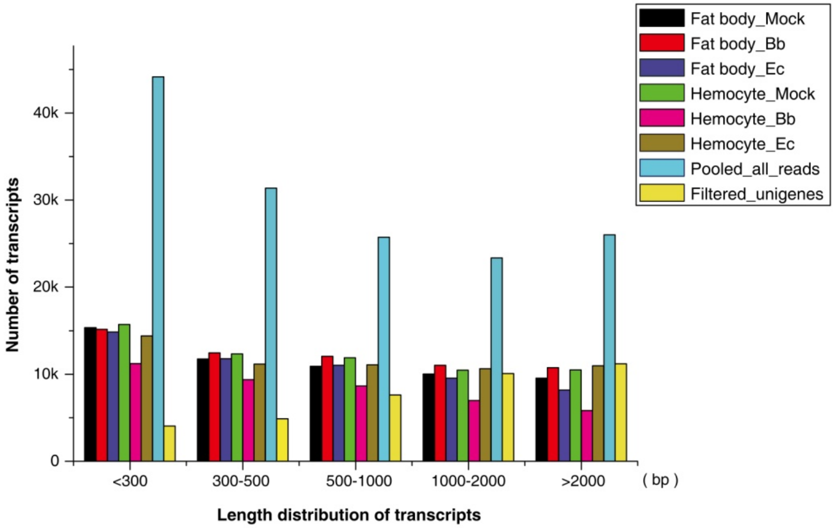
Fat body_Mock
Fat body_Bb
Fat body_Ec
Hemocyte_Mock
Hemocyte_Bb
Hemocyte_Ec
Pooled_all_reads
Filtered_unigenes
Number of transcripts
40k
30k
20k
10k
0
<300
300-500
500-1000
1000-2000
>2000
(bp)
Length distribution of transcripts

B

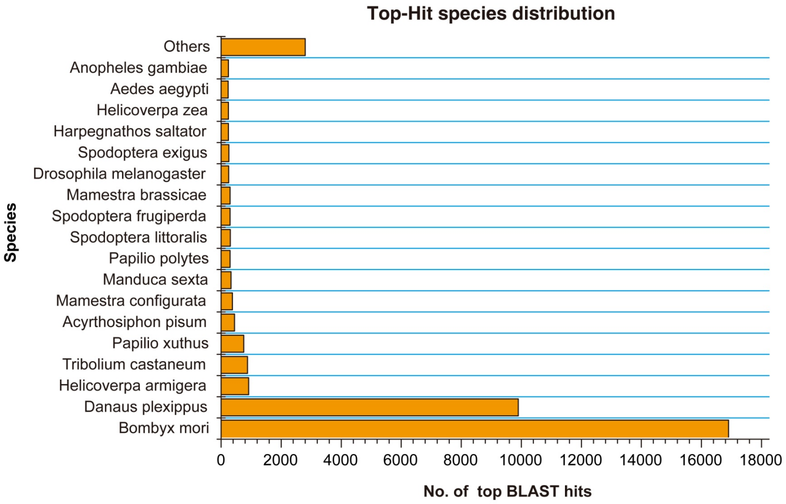
Top-Hit species distribution
Species
Others
Anopheles gambiae
Aedes aegypti
Helicoverpa zea
Harpegnathos saltator
Spodoptera exigus
Drosophila melanogaster
Mamestra brassicae
Spodoptera frugiperda
Spodoptera littoralis
Papilio polytes
Manduca sexta
Mamestra configurata
Acyrthosiphon pisum
Papilio xuthus
Tribolium castaneum
Helicoverpa armigera
Danaus plexippus
Bombyx mori
0
2000
4000
6000
8000
10000
12000
14000
16000
18000
No. of top BLAST hits

Fig. S4

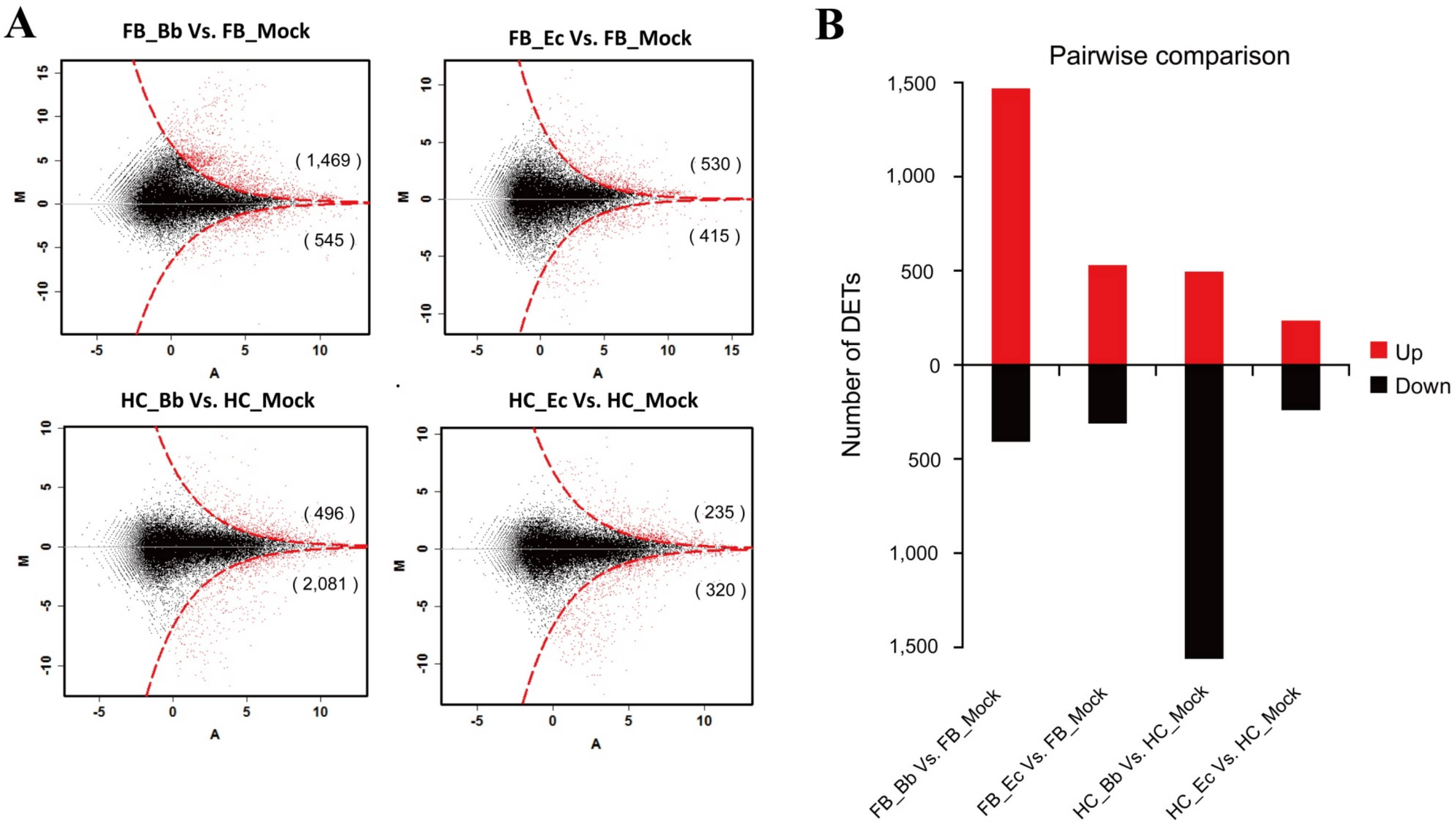
A
FB_Bb Vs. FB_Mock
(1,469)
(545)
FB_Ec Vs. FB_Mock
(530)
(415)
HC_Bb Vs. HC_Mock
(496)
(2,081)
HC_Ec Vs. HC_Mock
(235)
(320)
M
A
B
Pairwise comparison
Number of DETs
Up
Down
FB_Bb Vs. FB_Mock
FB_Ec Vs. FB_Mock
HC_Bb Vs. HC_Mock
HC_Ec Vs. HC_Mock

Fig. S5

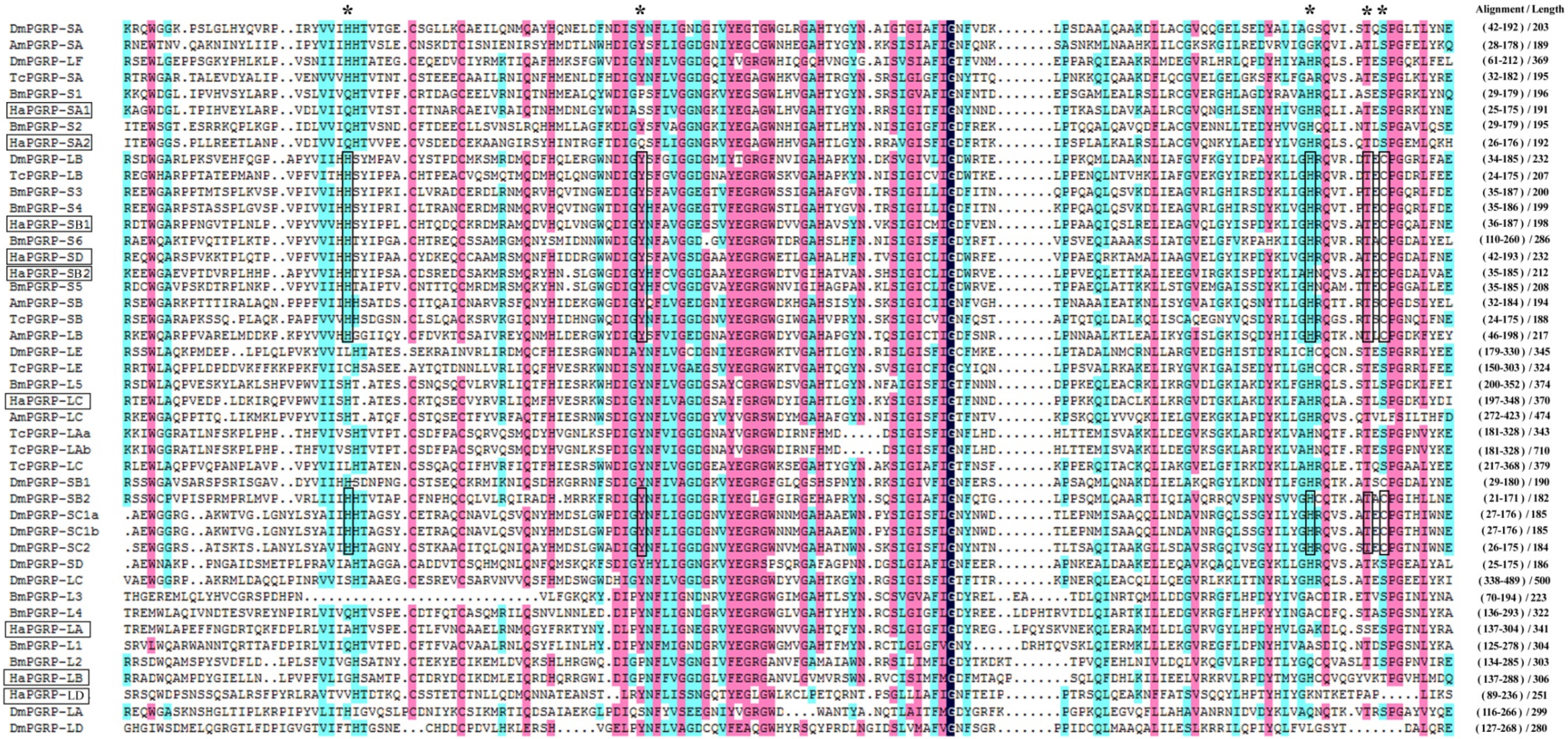
Alignment / Length
DmPGRP-SA (42-192) / 203
AmPGRP-SA (28-178) / 189
DmPGRP-LF (61-212) / 369
TcPGRP-SA (32-182) / 195
BmPGRP-S1 (29-179) / 196
HaPGRP-SA1 (25-175) / 191
BmPGRP-S2 (29-179) / 195
HaPGRP-SA2 (26-176) / 192
DmPGRP-LB (34-185) / 232
TcPGRP-LB (24-175) / 207
BmPGRP-S3 (35-187) / 200
BmPGRP-S4 (35-186) / 199
HaPGRP-SB1 (36-187) / 198
BmPGRP-S6 (110-260) / 286
HaPGRP-SD (42-193) / 232
HaPGRP-SB2 (35-185) / 212
BmPGRP-S5 (35-185) / 208
AmPGRP-SB (32-184) / 194
TcPGRP-SB (24-175) / 188
AmPGRP-LB (46-198) / 217
DmPGRP-LE (179-330) / 345
TcPGRP-LE (150-303) / 324
BmPGRP-L5 (200-352) / 374
HaPGRP-LC (197-348) / 370
AmPGRP-LC (272-423) / 474
TcPGRP-LAa (181-328) / 343
TcPGRP-LAb (181-328) / 710
TcPGRP-LC (217-368) / 379
DmPGRP-SB1 (29-180) / 190
DmPGRP-SB2 (21-171) / 182
DmPGRP-SC1a (27-176) / 185
DmPGRP-SC1b (27-176) / 185
DmPGRP-SC2 (26-175) / 184
DmPGRP-SD (25-175) / 186
DmPGRP-LC (338-489) / 500
BmPGRP-L3 (70-194) / 223
BmPGRP-L4 (136-293) / 322
HaPGRP-LA (137-304) / 341
BmPGRP-L1 (125-278) / 304
BmPGRP-L2 (134-285) / 303
HaPGRP-LB (137-288) / 306
HaPGRP-LD (89-236) / 251
DmPGRP-LA (116-266) / 299
DmPGRP-LD (127-268) / 280

Fig. S6

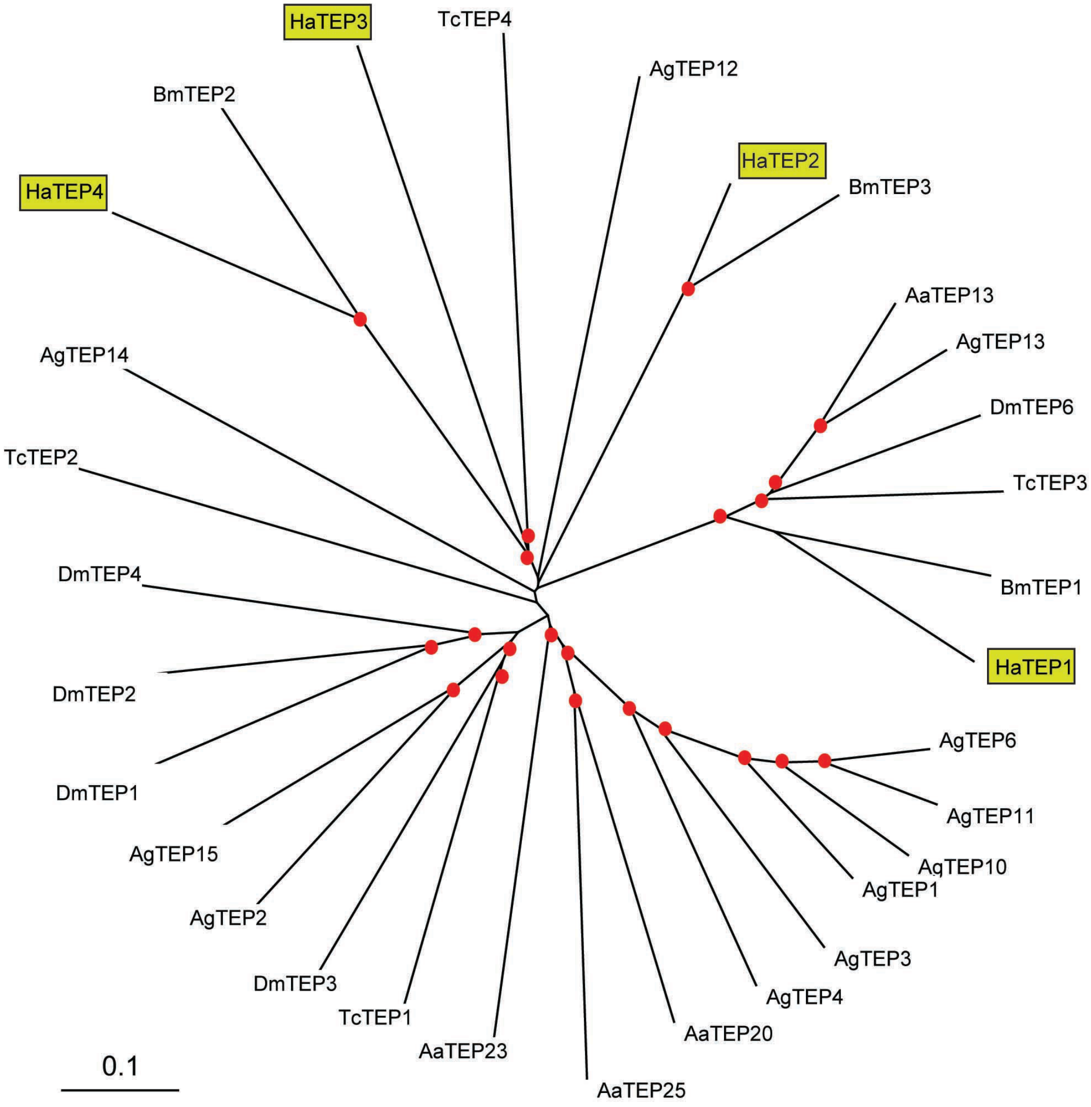
HaTEP3
TcTEP4
AgTEP12
BmTEP2
HaTEP2
BmTEP3
HaTEP4
AaTEP13
AgTEP13
AgTEP14
DmTEP6
TcTEP2
TcTEP3
DmTEP4
BmTEP1
HaTEP1
DmTEP2
AgTEP6
DmTEP1
AgTEP11
AgTEP10
AgTEP1
AgTEP15
AgTEP2
AgTEP3
AgTEP4
DmTEP3
TcTEP1
AaTEP20
AaTEP23
AaTEP25
0.1

Fig. S7

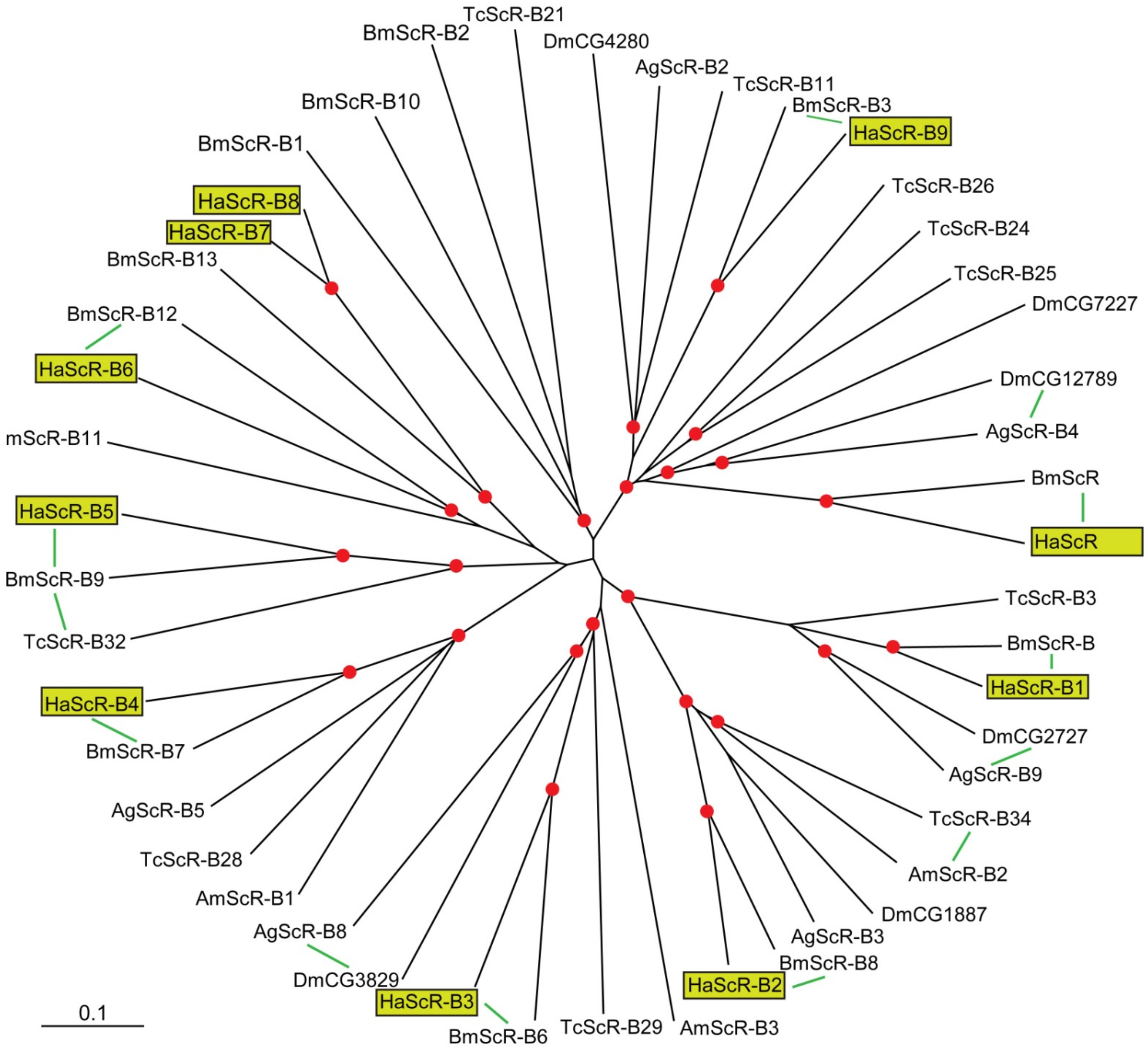
TcScR-B21
BmScR-B2
DmCG4280
AgScR-B2
TcScR-B11
BmScR-B3
HaScR-B9
BmScR-B10
BmScR-B1
TcScR-B26
HaScR-B8
HaScR-B7
TcScR-B24
BmScR-B13
TcScR-B25
DmCG7227
BmScR-B12
HaScR-B6
DmCG12789
mScR-B11
AgScR-B4
BmScR
HaScR-B5
HaScR
BmScR-B9
TcScR-B3
TcScR-B32
BmScR-B
HaScR-B1
HaScR-B4
BmScR-B7
DmCG2727
AgScR-B9
AgScR-B5
TcScR-B34
TcScR-B28
AmScR-B2
AmScR-B1
DmCG1887
AgScR-B8
AgScR-B3
BmScR-B8
DmCG3829
HaScR-B2
HaScR-B3
BmScR-B6
TcScR-B29
AmScR-B3
0.1

Fig. S8

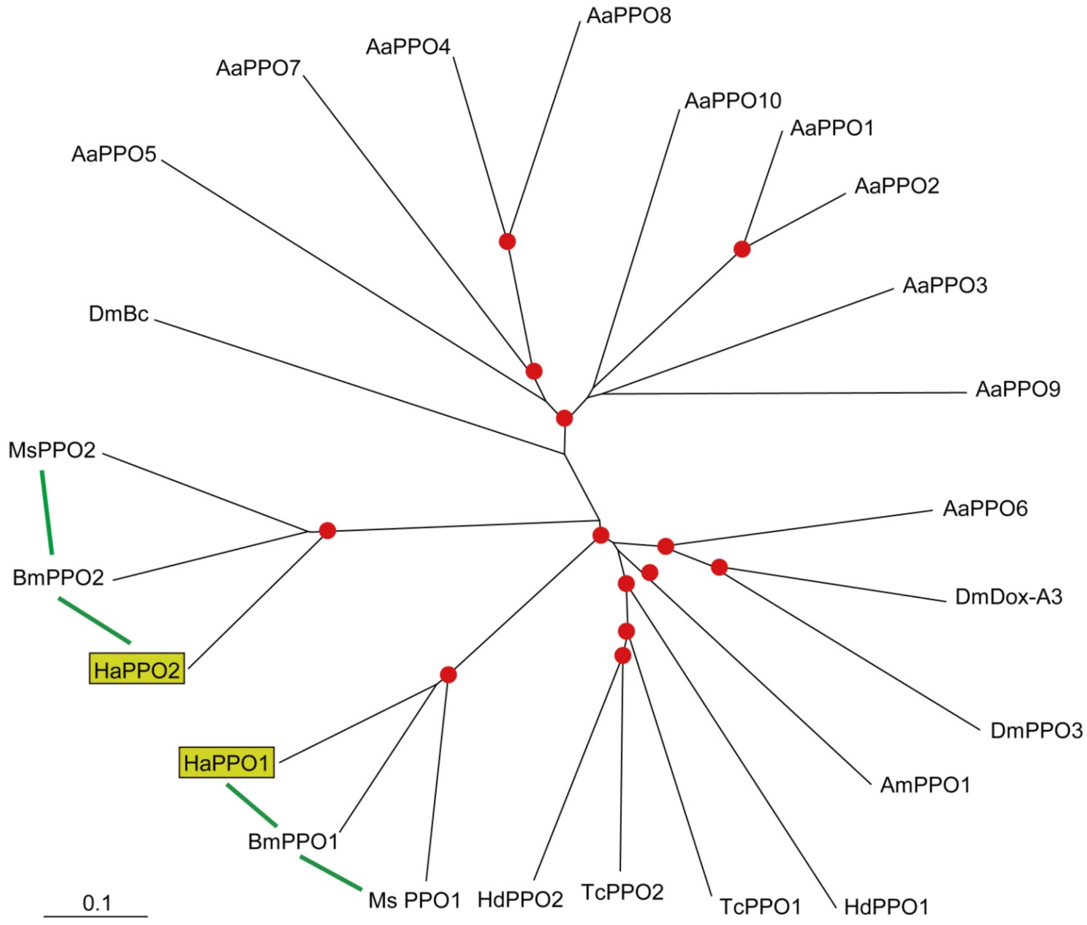
AaPPO8
AaPPO4
AaPPO7
AaPPO10
AaPPO1
AaPPO5
AaPPO2
AaPPO3
DmBc
AaPPO9
MsPPO2
AaPPO6
BmPPO2
DmDox-A3
HaPPO2
DmPPO3
HaPPO1
AmPPO1
BmPPO1
0.1
Ms PPO1
HdPPO2
TcPPO2
TcPPO1
HdPPO1

Fig. S9

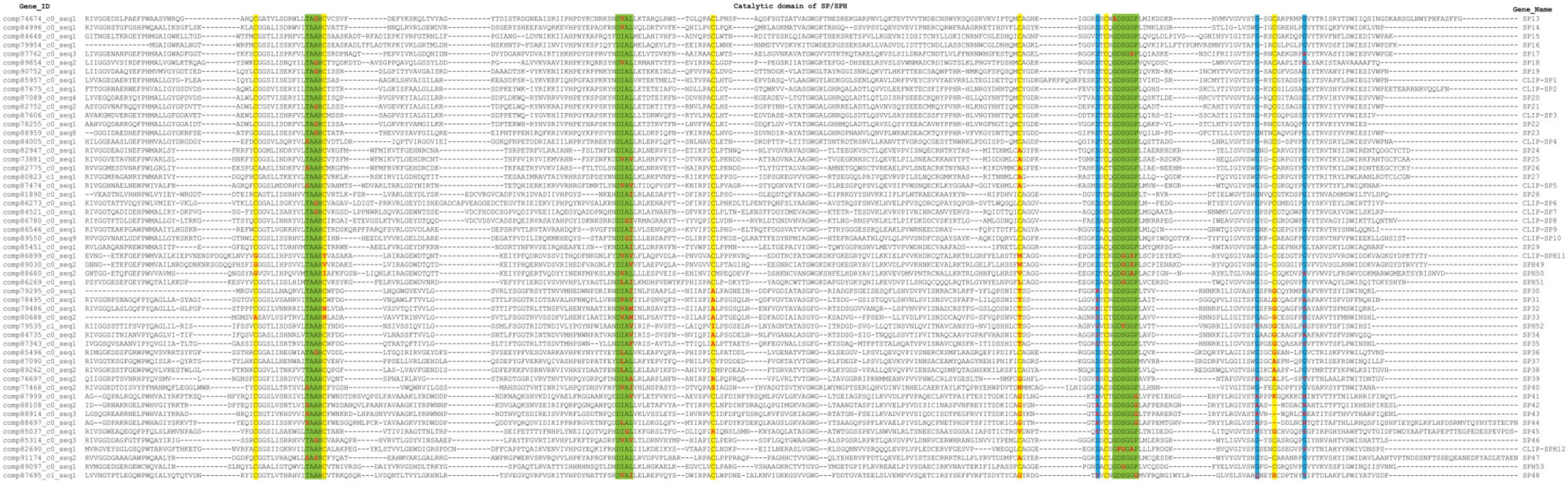

Fig. S10

```
Serpin         P14                P1                     P12'
                |                  |                       |
1        NEEGAEAAAANGVFTMFRR----RIN----PKIFNVDRPFFFA
2        NEEGAEAAAATAMGISTLS----AMISFEPVPSFVADRPFLAI
4        TEEGTEAAGVTAAEFSNR----------IGVVQFQANRPFTYM
5        TESGTVASAVSTATFSDR----------ISAPYFHANRPFIYF
9        TEDGTVAAAATAALFESR----------MLPEQFVANKPFMFM
12       NEKGVSAYAGSLVIKDNV-------------TEFNCNRPFAYM
17       DNHGVYSSPFEVPQATPYVAEGPTGLDPKVGNEFIADRPFLFA
13       NNTGLYFEPAEYSHPSGLR----TGLEPILGTDFIADRPFLFG
15       DNQGIRPAPATTDALVNV----TTNIDPVVGREFVANRPFLFG
18       NKDGVNQPAVVELRSFG----PPSSPEDQFLSEFVADRPFMFG
11       DEKGTEAAAATAIIVGLTSAY-----PPPKTYIFKATHPFAFF
22       NEEGTVAAAATIVGLEDR----------ILGQRFEANKEFIFL
8        DEEGATAGAFTGLFSFKL-------------------------
16       DELGLGDKQDIDFQDPEEIP--DSPSRPRVPQLFKADHAFAYY
3        NELGSVAYSATEISLVNKFG-----EDTEAYAEVVANKPFLFF
6        DEEGTVAAAATAIFGFRSSR-------PAEPSRFIANFPFVYL
7        DEKGGSAAAATAFAAVALSY-------DEPSIRFRVNRPFIAV
19       DETGTIAAASTAGMVVPLIF---------SQVQLRVDRPFVFF
20       DDADSSASQIAAANPVMEMSKNLPLASSKPLRKFNVEHPFIFY
21       GEDGVDVGDAMHLPLHLRPRQARLPARSALPARLKFDRPFLYF
14       PESPEDPHGDNENKENQQEKDVEVKDEGSNKTETKVTEVQKTD
10       DEPTT--------------------------NNEVDTSPTTVD
```

Fig. S11

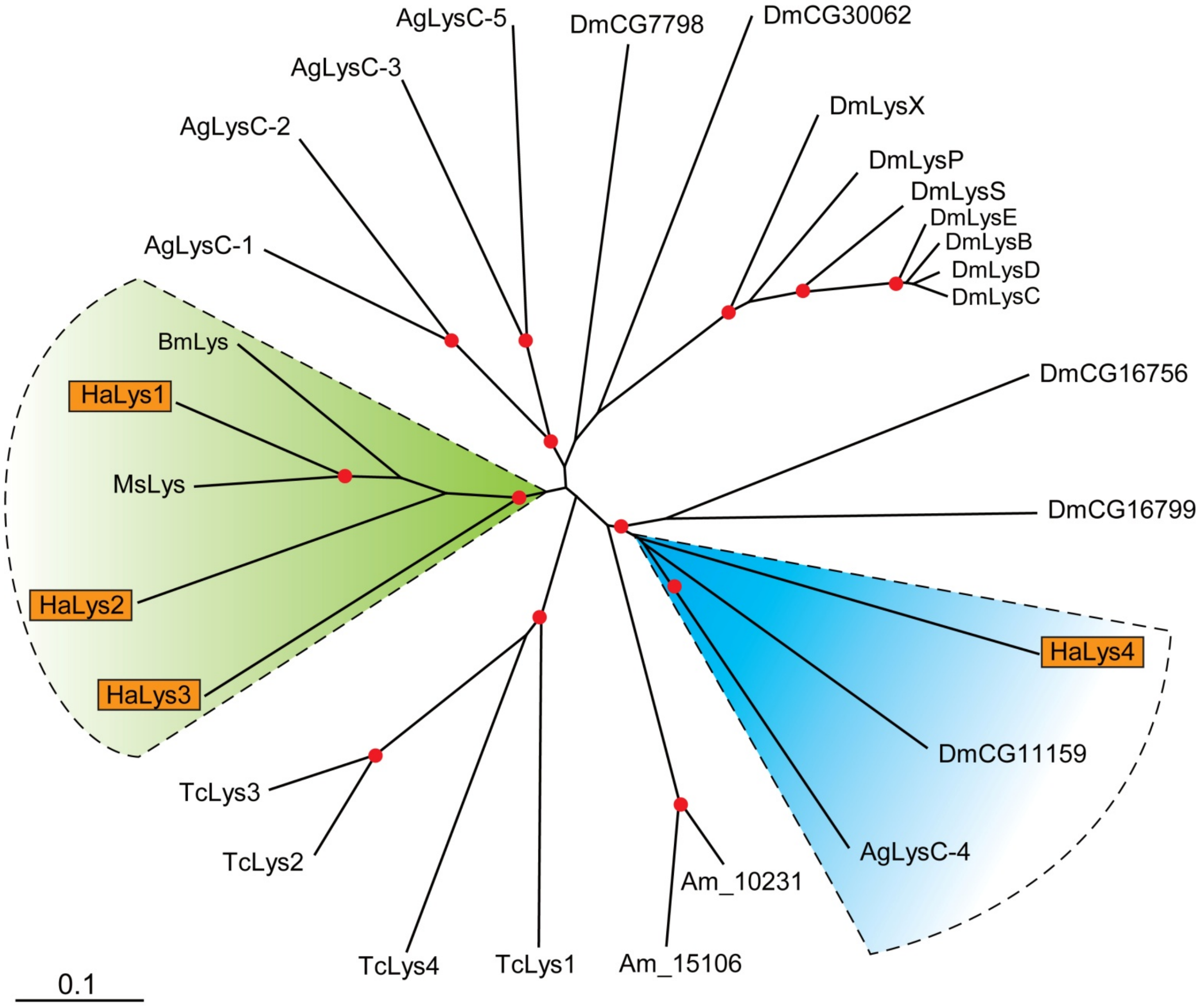
AgLysC-5
DmCG7798
DmCG30062
AgLysC-3
DmLysX
AgLysC-2
DmLysP
DmLysS
DmLysE
AgLysC-1
DmLysB
DmLysD
DmLysC
BmLys
DmCG16756
HaLys1
MsLys
DmCG16799
HaLys2
HaLys4
HaLys3
DmCG11159
TcLys3
AgLysC-4
TcLys2
Am_10231
TcLys4
TcLys1
Am_15106
0.1

Fig. S12

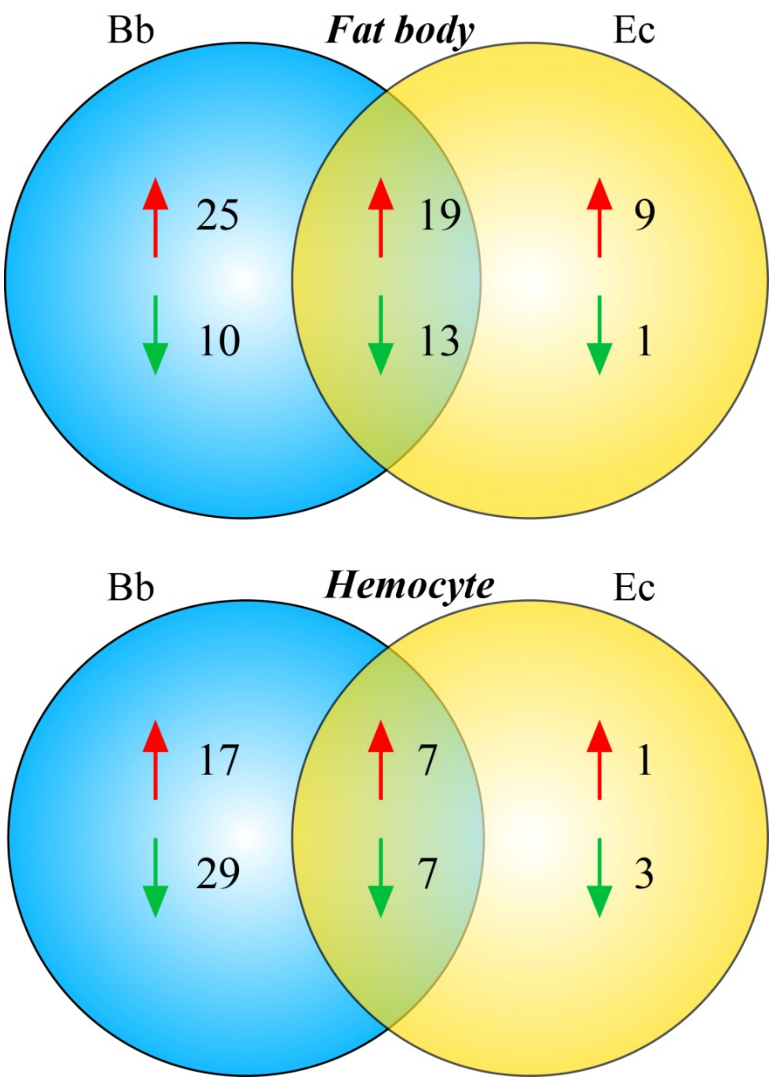

Supplement: Additional file 1: Figure S1. — Morphological characteristics of microbial pathogens. (A) The entomopathogenic fungi were separated into conidia (blue arrowhead) and hyphae (red arrowhead). (B) The rod-shaped bacteria E. cloacae were colored light pink (red arrowhead) after Gram staining. Figure S2. Flowchart of the RNA-seq analysis. Figure S3. Length distribution of the H. armigera cDNA contigs and their comparison with homologs in other insect species. (A) Length distribution of the contigs assembled using different strategies. (B) Comparison of the initially obtained unigenes with homologous sequences in other insects. Figure S4. Identification of DETs in the H. armigera immunotranscriptome. (A) The MA-plot displays the DETs identified from different comparisons. M is the Y-axis representing the intensity ratio, and A is the X-axis representing the average intensity for each transcript. (B) Comparison of DEGs in hemocytes and fat body infected by B. bassiana and E. cloacae. Figure S5. Multiple sequence alignment of PGRP domains. Figure S6. Phylogenetic analysis of the thioester proteins in H. armigera and other insects. Figure S7. Phylogenetic analysis of the scavenger receptor Bs in H. armigera and other insects. Figure S8. Phylogenetic analysis of prophenoloxidases in H. armigera and other insects. Figure S9. Sequence alignment of the catalytic domains in SPs and SPHs. The green and yellow shades indicate the conserved residues. Determinants of the primary substrate-binding pocket are shade cyan. Figure S10. Reactive site loops of the H. armigera serpins. Gap positions are manually adjusted for better alignment. Predicted P1 residues are shown in bold and red. Figure S11. Phylogenetic analysis of the lysozymes in H. armigera. Figure S12. Venn diagram showing the unique and shared immunity-related DETs in fat body and hemocytes after the fungal and bacterial infection. The overlapping regions represent genes concomitantly regulated under the two experimental conditions. [file 12864_2015_1509_MOESM1_ESM.pdf]
